# HPRT-Deficiency Dysregulates cAMP-PKA Signaling and Phosphodiesterase 10A Expression: Mechanistic Insight and Potential Target for Lesch-Nyhan Disease?

**DOI:** 10.1371/journal.pone.0063333

**Published:** 2013-05-14

**Authors:** Ghiabe-Henri Guibinga, Fiona Murray, Nikki Barron

**Affiliations:** 1 Department of Pediatrics, Division of Genetics, University of California San Diego, School of Medicine, La Jolla, California, United States of America; 2 Departments of Medicine & Pharmacology, University of California San Diego, School of Medicine, La Jolla, California, United States of America; University of Oslo, Norway

## Abstract

Lesch-Nyhan Disease (LND) is the result of mutations in the X-linked gene encoding the purine metabolic enzyme, hypoxanthine guanine phosphoribosyl transferase (HPRT). LND gives rise to severe neurological anomalies including mental retardation, dystonia, chorea, pyramidal signs and a compulsive and aggressive behavior to self injure. The neurological phenotype in LND has been shown to reflect aberrant dopaminergic signaling in the basal ganglia, however there are little data correlating the defect in purine metabolism to the neural-related abnormalities. In the present studies, we find that HPRT-deficient neuronal cell lines have reduced CREB (cAMP response element-binding protein) expression and intracellular cyclic AMP (cAMP), which correlates with attenuated CREB-dependent transcriptional activity and a reduced phosphorylation of protein kinase A (PKA) substrates such as synapsin (p-syn I). Of interest, we found increased expression of phosphodiesterase 10A (PDE10A) in HPRT-deficient cell lines and that the PDE10 inhibitor papaverine and PDE10A siRNA restored cAMP/PKA signaling. Furthermore, reconstitution of HPRT expression in mutant cells partly increased cAMP signaling synapsin phosphorylation. In conclusion, our data show that HPRT-deficiency alters cAMP/PKA signaling pathway, which is in part due to the increased of PDE10A expression and activity. These findings suggest a mechanistic insight into the possible causes of LND and highlight PDE10A as a possible therapeutic target for this intractable neurological disease.

## Introduction

Mutations in the gene encoding the purine biosynthetic enzyme Hypoxanthine phosphoribosyltransferase (HPRT) (IMP: pyrophosphate Phosphoribosyltransferase; EC 2.4.2.8) leads to both metabolic and neurological defects that can lead to Lesch-Nyhan Disease (LND). The impairment in purine metabolism associated with LND has been well characterized and recognized clinically as hyperuricemia, which can be treated with allopurinol. However, other features of LND such as dystonia, choreoathetosis, mental retardation and the hallmark neurobehavioral trait of compulsive self-mutilation are mostly untreatable [1]. Post-mortem analysis of LND patients and studies of HPRT-knock out (KO) mice have indicated that dysfunctional dopaminergic signaling in the midbrain and the basal ganglia may cause this disease phenotype, although the mechanisms underlying the pathogenesis of LND are not well understood [2]. HPRT-deficiency has been shown to alter the expression of a number of transcription factors and key signaling components that are necessary for neuronal development, however these data still do not fully elucidate the relationship between the defect in the purine metabolism and the neural phenotype associated with LND [3]–[6]. For the current study, we hypothesize that altered purine metabolism due to HPRT-deficiency affects the homeostasis of signaling pathways related to purine metabolic functions, including ubiquitously expressed second messengers such as cyclic AMP (cAMP). We have previously shown that HPRT-deficiency leads to the dysregulation of microRNA-181a (miR-181a) [7], here we have carried out supplemental analysis of miR-181a target genes using gene ontology analysis, and uncovered genes implicated in the regulation cAMP/PKA signaling pathway. Our data show that HPRT-deficiency leads to a reduced expression of CREB, blunted cAMP production and reduced phosphorylation of PKA substrates, including phospho-synapsin, in HPRT-deficient MN9D neuronal cell lines. Furthermore, we identified increased PDE10 expression in HPRT-deficient cells which contributes at least in part to the decreased cAMP/PKA signaling. Overall, our data provide a mechanism by which blunted cAMP/PKA signaling and phosphorylation of PKA substrates, such as synapsin, may contribute to the neurological phenotype associated with HPRT-deficiency and also highlights PDE10 as a potential target for LND.

## Materials and Methods

### Cells

Human SH-SY5Y cells (ATCC) were maintained in a 1∶1 mixture of Eagle’s minimum essential medium and F12 Medium (Gibco, Carlsbad CA) containing 10% fetal bovine serum (FBS) and 50 µg/ml penicillin/streptomycin (Invitrogen, Carlsbad, CA) in 5% CO_2_. Parent HPRT positive cells and HPRT deficient mutant MN9D cells were obtained from Dr. Jinnah (Emory University, Atlanta, GA) [8]. MND9 and Human embryonic kidney (HEK, ATCC) 293 cells were cultured at 37°C under in 5% CO_2,_ in DMEM medium supplemented with 10% FBS, 50 µg/ml penicillin/streptomycin. We also selected human control (CTL), HPRT-deficient fibroblasts consistent with partial (LNV) or complete (LND) HPRT-enzymatic activity. LNV and LND phenotypes represent mildly and severely affected patients, respectively. These fibroblasts were also kindly provided by Dr. Jinnah (Emory University, Atlanta, Ga), and grown in DMEM medium supplemented with 10% FBS, 50 µg/ml penicillin/streptomycin.

### HPRT and Luciferase Short Hairpin Oligonucleotides and Knockdown

Short hairpin RNA (shRNA) sequences against the luciferase and HPRT genes were prepared and transfected as previously described [4] [7]. HEK293 cells were infected at a multiplicity of infection (MOI) of approximately 1 with the knockdown lentivector-sh2hprt (directed against HPRT) or with control lentivector-shlux (directed against luciferase) as previously described [9], [10].

### Total RNA Isolation and Quantitative PCR Analysis

Total RNA was isolated for QPCR as previously described [7]. Table S1 lists the primers used in this study.

### cAMP Assay

Control and HPRT-deficient cells were seeded in serum free DMEM at a density of 2×10^5^ per cm^2^, and then treated with vehicle (1% DMSO in PBS) or 1–50 µM of forskolin (SIGMA) for 15 min. In other conditions, cells were pre-treated for 30 min before the addition of forskolin with 50–200 µM of PDE10A inhibitor Papaverine, or with 30 µM of PDE4 inhibitor, Rolipram or with 30 µM of PDE1 inhibitor, Vinpocetin or 30 µM of PDE7 inhibitor, BRL5081 (all from Sigma) or vehicle. Reaction was terminated by removal of the medium and the subsequent addition of 0.1M HCl. cAMP accumulation was measured using “cAMP complete ELISA kit” from Enzo-Life Sciences (Cat# ADI-900-163). Samples were treated according to the manufacturer instructions, and the amount of cAMP was normalized to protein content. Alternatively, the cAMP level in cells was measured using radioimmunoassay and normalized to the amount of protein per well as previously described [11], [12].

### CRE-reporter & Immunocytochemistry

Control or HPRT-knockdown HEK 293 cells were seeded at density 2×10^5^ cells per cm^2^ and transfected after 24 hr with pCRE-DD-Zs-Green1 plasmid, a reporter that allows the measurement of cAMP response element binding protein (CREB) activity (details of the reporter assay can be found at www.clontech.com cat No 631085). Thirty six hours later, CRE-DD-Zs-Green1 transfected control and HPRT-deficient cells were then stimulated with forskolin (50 µM) as described above, in the presence of 1 µM of shield1. Cells were fixed with 4% paraformaldehyde and treated with 0.2% Triton-X-100 in 3% horse serum for 15 minutes and then blocked for one hour in 3% horse serum in PBS. The cells were then incubated for 15 minutes in 0.01% of 4′,6-diamidino-2-phenylindole (DAPI) (Invitrogen) for nuclear staining. The resulting cells were mounted with Vectashield media (Vector Laboratories, Burlingame, CA). Fluorescence was visualized using Olympus BX51 fluorescent microscope with BP72 Olympus acquisition camera. Images were captured for each experimental condition and green fluorescence quantified by mean fluorescent unit using Image J software.

### Protein Kinase A (PKA) Assay

Control and HPRT-deficient cells were seeded and treated with DMSO and forskolin as indicated above. The reaction was terminated by removal of the medium and subsequent addition of mammalian protein extraction reagent (M-PER from Thermo-Scientific) containing a cocktail of protease inhibitors (From Sigma). PKA activity was measured using “PKA kinase activity ELISA kit” from Enzo-Life Sciences and normalized to protein (Cat# ADI-EKS-390A).

### Immuno-blot Analysis

Cells were treated as indicated above and lysed using mammalian protein extraction reagent (M-PER from Thermo-Scientific) containing the protease inhibitor mixture, 1 mM PMSF, 1 mM, sodium orthovanadate (Santa Cruz Inc). The cell lysates were centrifuged 15,000 g at 4°C for 10 minutes and prepared for immuno-blot analysis with the following primary antibodies used at dilutions ranging from 1∶500 to 1∶1000 and incubated overnight at 4°C: The primary polyclonal rabbit antibodies against synapsin I, phospho-synapsin I (Ser9), GAPDH was obtained from (Cell Signaling Technology®, CST). Phospho-PKA substrate (RRXS*/T*) (100G7E) rabbit antibody was also obtained from CST. Additionally, primary rabbit antibodies against PDE4B, PDE10A and HPRT were obtained from Abcam. Goat and rabbit antibodies against PDE7B, β-actin and PDE1C were obtained from GeneTex, Santa Cruz and Fabgennix, respectively and secondary IgG antibodies labeled with horseradish peroxidase from Santa Cruz (dilution 1∶20000 incubated for one hour at room temperature). Western-blot signal was quantified using densitometry Image J software according to the protocol published at http://openwetware.org/wiki/Bitan:densitometry. β-actin or GAPDH were used as loading controls.

### 6-Bnz-cAMP Analog Treatment Assay

control and HPRT-deficient cells seeded in conditions indicated above were treated with vehicle PBS (control) and up to 200 µM of N6-Benzoyl adenosine-cAMP for 30 min in serum free conditions. The reaction was terminated by removal of the medium and subsequent addition of mammalian protein extraction reagent (M-PER from Thermo-Scientific) and a cocktail of protease inhibitors (From Sigma). Cell lysates were processed for immuno-blot analysis as described previously.

### siRNA Experiments

2×10^5^ HPRT-deficient MN9D cells in 6 well-plate were exposed to 80 pmole of control (scramble siRNA) or PDE10A siRNA (directed to mouse PDE10A) from Santa cruz biotechnology and transfected according to the established transfection protocol (http://datasheets.scbt.com/siRNA_protocol.pdf). Forty eight hours after transfection, the cells were lysed and processed for protein quantification and immuno-blot analysis against PDE10 and GAPDH. The siRNA transfected cells (siRNA-CTL and siRNA-PDE10) were also treated with forskolin as indicated above.

### Lentivirus Preparation and HPRT-Reconstitution Experiment

Lentivirus-based plasmids expressing GFP and HPRT genes were generated by inserting cDNA for GFP (obtained by PCR from p-EGFP-N1 cloning vector, from Clontech), and cDNA from HPRT (from OriGene) into pSin-EF2-Puro (from Addgene) using SpeI and EcoRI restriction site. pSin-EF2-Puro contains a constitutively active promoter from human elongation factor and a puromycin resistance marker for selection of stable transfectants. VSV-G pseudotyped lentivirus–based vectors expressing GFP, as well as HPRT were prepared by using HEK 293T cells and were subjected to the established triple transduction protocol as previously described [9], [13]. HPRT-deficient MND9 cells were infected at the multiplicity of infection (MOI) of 100 and selected for puromycin for seven days (1 µg/ml). The HPRT phenotype of the infected cells was confirmed by growing the cells in 250 µM of 6-thioguanine (6-TG) (SIGMA) and in 1× hypoxanthine aminopterin thymidine (HAT) medium (from ATCC). The growth of the GFP-infected HPRT-deficient MN9D cells remains unaltered in 6-TG while it was severely impaired in HAT (data not shown). Conversely, HPRT-reconstituted MND9 cells were now displaying altered growth in 6-thioguanine (data not shown). The HPRT phenotype of MN9D infected cells was genotypically confirmed using PCR and Immuno-blot with an HPRT antibody (Abcam).

### Statistical Analysis

Statistical analyses were carried out using Kaleidagraph graphing & data analysis software package (Synergy Software, Reading Pa). The data are reported as mean +/− standard error (SE). Student paired t-tests were performed for control and experimental groups or One way ANOVA with Tukey post-hoc test where appropriate. Statistical significance was set at p<0.05.

## Results

### Mir-181a-mediated Regulation of cAMP/PKA Related Genes in HPRT-deficient Cells

We have previously demonstrated that HPRT-deficiency dysregulates the expression of various microRNAs, including miR-181a in SH-SY5Y human neuroblastoma cell lines, which in turn targets several neuro-developmental genes [7]. For this study, we submitted the list of miR-181a target genes to DAVID (Data annotation for visualization of integrated discovery) [14]–[16] in order to identify additional genes and pathways involved in the regulation of cyclic purine nucleotides pathway. Table S2 shows some GO terms derived from miR-181a target genes. Among the GO terms, the “*purine nucleotide metabolic process”* term contains the list of genes involved in the cAMP/PKA pathway, such as the transcription factor CREB that had previously been identified as a potential target of miR-181a [7].

### CREB-mediated Transcriptional Activity is Reduced in HPRT-knockdown (KD) Cells

**Figure 1:**
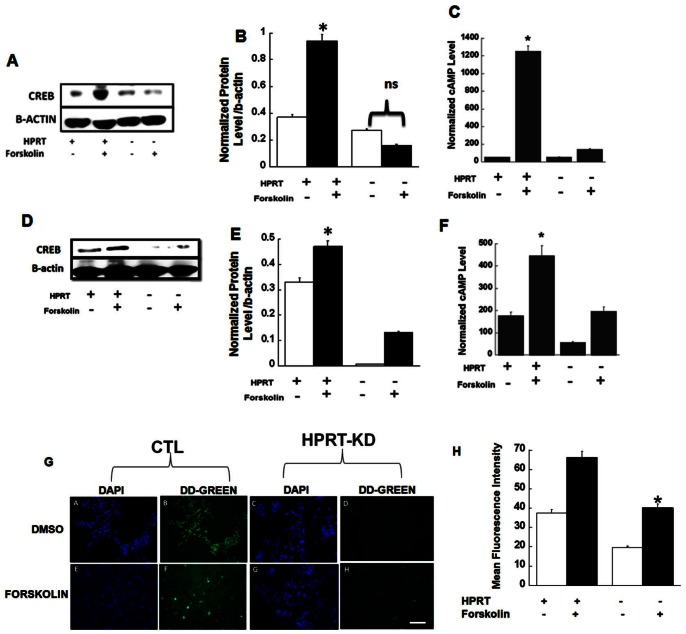
Reduction of CREB expression in HPRT-deficiency. Reduction of CREB expression in HPRT-deficient human SH-SY5Y (**A & B**) and mouse MN9D (**D & F**) cells lines. Cells were then stimulated with DMSO (as control) or Forskolin (see methods). Immuno-blot as well as the quantification of protein through densitometry analysis show impaired expression of CREB in response to forskolin. The asterisks (*) represent statistical significance between forskolin treated cells (p<0.05, t-test n = 3). Reduced agonist-induced cAMP accumulation in HPRT-deficient SH-SY5Y (**C**) and MN9D (**F**) cells. Cells were stimulated with DMSO (CTL) or forskolin. Cyclic AMP level was evaluated as described in material and methods. The data are expressed as level of cAMP normalized to protein content. Error bars represent mean ± SEM of triplicate measurements of two experiments (n = 6). The asterisks (*p<0.05) represent statistical significance between forskolin treated cells (t-test). (**G & H**) Altered-CREB-mediated transcriptional activity in HPRT-deficiency; HEK293 cells lines were infected with lentivirus vector encoding small hairpin against luciferase (HPRT+) and HPRT gene (HPRT−). Cells were subsequently transfected with pCRE-DD-Zs-Green1 (CREB probe) and then stimulated with DMSO (CTL) or 50 µM Forskolin for 30 min. Figure shows microscopy images of DAPI staining and green fluorescence which is a measure of the overall CREB-related transcriptional activity. There is diminished green fluorescence in HPRT-deficient cells relative to control cells after stimulation with forskolin. (Bar scale, 100 µm). This is confirmed by the quantification of the mean fluorescent intensity illustrated in Figure H. Error bars represent mean ± SEM of duplicate measurements of two independent experiments. The asterisks (*) represent statistical significance between forskolin treated cells (p<0.05, t-test).

To determine the role of CREB in HPRT-deficiency, we treated human (SH-SY5Y) and mouse (MN9D) control cells and HPRT-deficient cell lines with forskolin, a direct adenylyl cyclase activator that increases cAMP accumulation. We found forskolin increased CREB expression in control cells, but not HPRT-deficient cells (Fig. 1A & 1B for SH-SY5Y cells and Fig. 1D & 1E for MN9D cells). The decrease in response correlated to reduced cAMP accumulation in both human and mouse HPRT-deficient cell lines (Fig. 1C & Fig. 1F). To show further evidence that HPRT-deficiency blunts CREB expression, we used a pCRE-DD-Zs-Green reporter vector (CREB probe) to monitor CREB activation in control and HPRT-deficient human embryonic kidney (HEK) 293 cells (control, Lenti-shlux and HPRT-knockdown Lenti-sh2HRPT). The HPRT-knock-down in HEK293 was verified by western-blot analysis and show a significant knock-down of the HPRT gene (Fig. S1), additionally HEK293 HPRT-knock down cells were also able to grow in 6-thioguanine (data not presented). The CREB probe (CRE-DD-Zs plasmid) contains cyclic AMP response elements that bind CREB to induce the expression of DD-Zs-Green fluorescent gene. We found that CREB-dependent promoter activity as measured by the level of green fluorescence intensity was reduced by 50% in HPRT-knockdown HEK293 cells upon forskolin treatment (Fig. 1G & 1H). HEK293 HPRT-KD cells also produced less cAMP in response to forskolin (Fig. S2). Together these data show reduced cAMP accumulation and CREB expression and CREB-transcriptional activity in a variety of HPRT-deficient cells.

### HPRT-deficiency Decreases Synapsin I mRNA

In these experiments and all the following ones we examined cAMP/PKA-related signaling principally in HPRT-mutant MN9D cell lines made HPRT-deficient by 6-thioguanine mutation/selection that present 0.4% of HPRT enzyme activity [8]. These neuronal cell lines of a dopaminergic lineage have been used to evaluate dopamine related signaling and function in HPRT-deficiency and other neurological diseases that affect dopaminergic neurotransmitter system [17], [18]. Therefore, these cell lines are faithful surrogate model for studying the effects of purine metabolism deficit caused by HPRT-deficiency on neuronal signaling functions.

**Figure 2:**
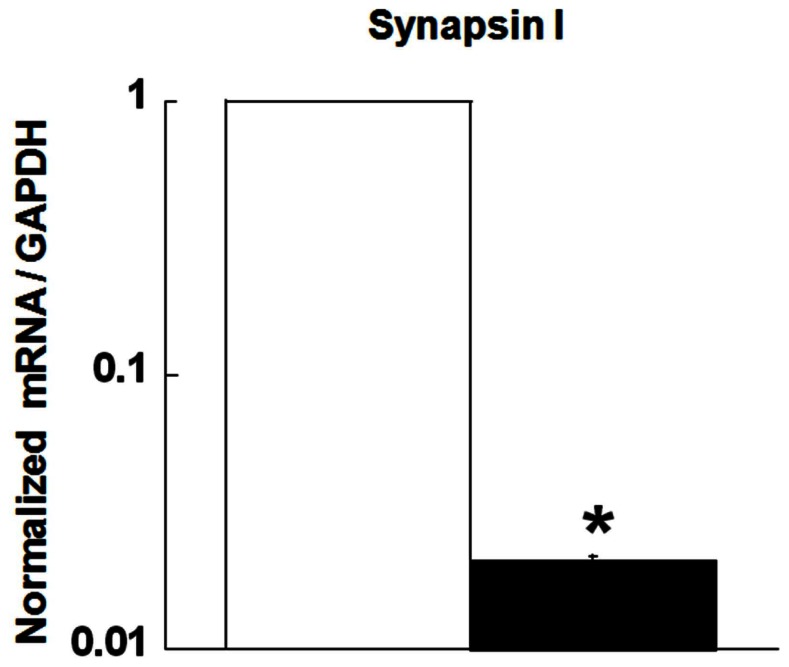
Reduction of Synapsin I mRNA level in HPRT-deficient cells. Synapsin I mRNA expression is reduced in HPRT-Deficient (mutant) MN9D cells. The asterisks (*) represent statistical significance between control (open bar) and mutant cells (closed bar) (n = 4, p<0.05, t-test).

The level of HPRT activity in these cells is metabolically consistent with the LND phenotype [8]. CREB binds DNA sequences with cAMP response elements (CRE), which are present in the promoter of many genes, including tyrosine hydroxylase (TH) and synapsin I [19], [20]. Studies from our laboratory and other groups have previously reported decreased TH mRNA levels in HPRT-deficient cells [4], [8]. In the current study, we demonstrate a significant reduction of Synapsin I mRNA level in HPRT-deficient (mutant) cell lines compared to control (Fig. 2 p<0.05). These results support the conclusion that HPRT-deficiency reduces CREB-related transcriptional activity.

### HPRT-deficiency Attenuates PKA Activity

**Figure 3:**
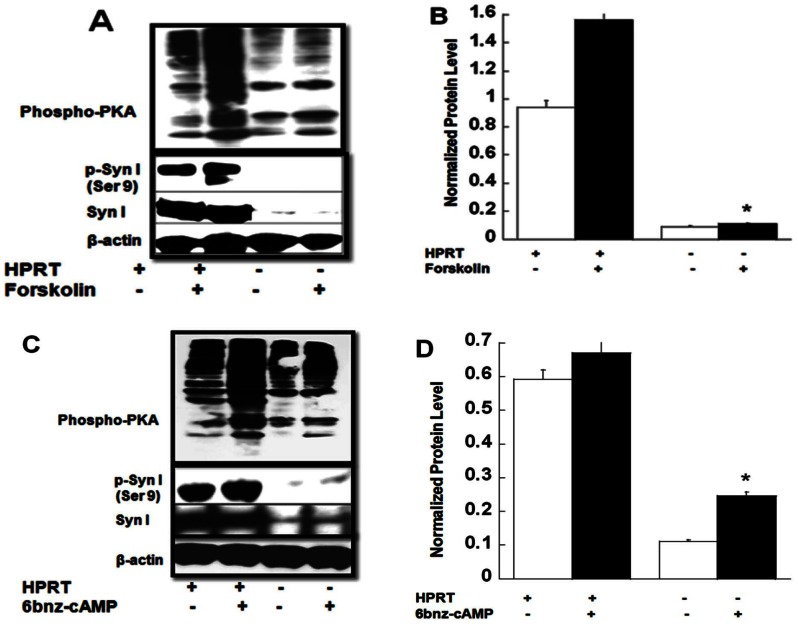
Blunted PKA-mediated signaling in HPRT-deficient MN9D cells. (**A**), Immuno-blot analysis of phospho-PKA-substrate expression and p-Syn I (Ser9) in response to forskolin exposure. (**B**), quantification phospho-synapsin relative to the total amount of protein measured as beta-actin. Error bars represent mean ± SEM of duplicate measurements of two independent experiments (n = 4). The asterisk (*) represents statistical significance between forskolin treated cells (p<0.05 t-test). (**C&D**), Effect of 6-bnz-cAMP on phospho-PKA-substrate and p-Syn I (Ser9) expression in HPRT-deficient MN9D cells. Data show reduced expression of phospho-PKA-substrates including p-Syn I (Ser9) in HPRT-deficient MN9D cells, after 6-bnz-cAMP treatment. The quantification of p-Syn relative to beta-actin is seen in figure D. Error bars represent mean ± SEM of duplicate measurements of two independent experiments (n = 4). The asterisk (*) represents statistical significance between 6-bnz-cAMP treated cells (p<0.05 t-test).

In order to unravel the impact of reduced cAMP accumulation on additional down-stream signaling effectors, we measured PKA expression and activity in HPRT-deficient MN9D cells. We used a phospho-PKA substrate (RRXS*/T*) (100G7E) monoclonal antibody to identify PKA substrates and evaluate the global pattern of expression of PKA substrates in cells. This antibody detects peptides and protein containing phospho Ser/Thr residues with arginine at the −3 and −2 positions. Figure 3A shows a global reduction of the activity of PKA-substrates in response to forskolin in HPRT-deficient MN9D cells compared to control, which corresponds to decreased PKA activity (Fig. S3). PKA is known to phosphorylate several protein substrates relevant to neural functioning, including Synapsin I [21]–[23]. Figures 3A & 3B demonstrate that there is significantly less phosphorylated Syn I (Ser9) protein in HPRT-deficient MN9D cells after forskolin treatment compared to control. Our data also show that HPRT-deficient MN9D cells display reduced global pattern of PKA-mediated phosphorylation in response to the PKA-selective cell permeable cAMP analog, N6-Benzoyladenosine-3′,5′-cyclic monophosphate (6-bnz-cAMP, 200 µM) (Fig. 3C); in addition, the level of phospho-synapsin expression is only marginally increased compared to control cells (Fig. 3C & 3D). To show whether the reduction of PKA-mediated synapsin I phosphorylation is only true for HPRT-deficient MN9D cells, we also carried out analysis of phospho-synapsin in HPRT-deficient human SH-SY5Y cells. Our data show blunted expression in response to forskolin can also be seen in SH-SY5Y (Fig. S4).

### Increased of Phosphodiesterase 10 (PDE10) Expression with HPRT-deficiency

**Figure 4:**
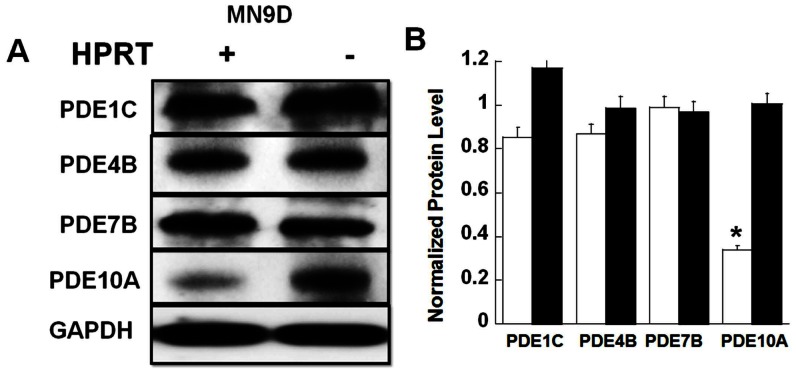
Increased PDE10A protein expression in HPRT-deficient MN9D cells. (**A&B**) Immuno-blot and quantification analysis for various PDEs that can affect cAMP/PKA signaling. Data show that the expression of PDE1C, PDE4B, and PDE7B are similar between control (parent) and HPRT-deficient (mutant) MN9D cell lines. Expression of PDE10A protein is significantly increased in HPRT-deficient cells. Error bars represent mean ± SEM of duplicate measurements of two independent experiments (n = 4). The asterisk (*) represents the statistical significant between the control (open bars) and HPRT-deficient (closed bars) MND9 cells (p<0.05 t-test).

**Figure 5:**
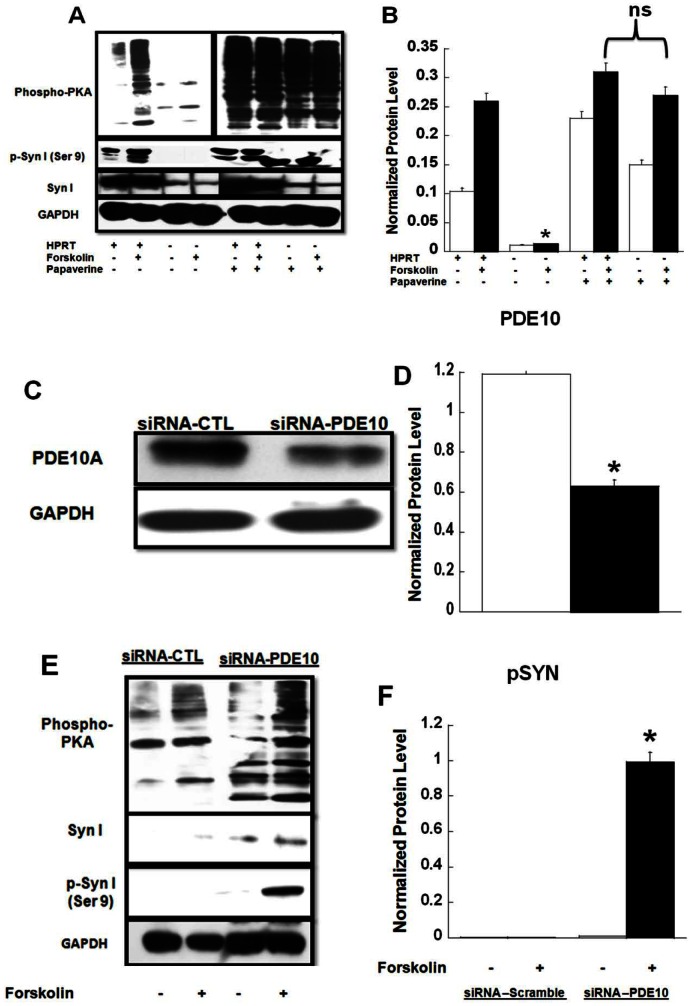
PDE10 inhibition restores PKA-mediated expression. (**A & B**), immuno-blot and quantification analysis of p-Syn (Ser9), data show that the lower expression of phospho-PKA substrate and p-Syn (Ser9) in response to forskolin treatment in HPRT-deficient MN9D cells is restored in the presence of Papaverine (200 µM). Error bars represent mean ± SEM of duplicate measurements of two independent experiments (n = 4). The asterisks (*) represent statistical significance between forskolin treated cells without papaverine treatment (p<0.05, t-test). (**C & D**), immuno-blot and quantification analysis of PDE10A expression after transfection of HPRT-deficient MN9D cells with siRNA directed to the mouse PDE10 gene. The data show lower expression PDE10A protein in cells transfected with siRNA-PDE10A relative to cells transfected with scramble control siRNA (siRNA-CTL). Error bars represent mean ± SEM of duplicate measurements carried out independently twice (n = 4). The asterisks (*) represent the statistical significant (p<0.05, t-test) between the control (open bar, siRNA-CTL) and siRNA-PDE10A transfected cells (closed bar). This significant reduction of PDE10A protein contributes to the enhanced expression of phospho-PKA-substrates including p-Syn observed in (**E & F**). Error bars represent mean ± SEM of duplicate measurements of two independent experiments (n = 4). The asterisk (*) represents the statistical significant (p<0.05, t-test) between the siRNA-CTL and siRNA-PDE10A HPRT-deficient transfected MND9 cells treated with forskolin.

To identify possible mechanisms by which HPRT-deficient cells attenuate PKA-related signaling, we noted several genes that are potential targets for miR-181a could regulate the level of cAMP signaling, namely adenylyl cyclase (AC) 1 and 9 as well as PDE10A (see Table S3). Interestingly, PDEs, including PDE10A, have previously been shown to be highly expressed in the brain, and are implicated in other neurological diseases [24], [25]. To examine the possible role of PDE10 in the reduction of cAMP/PKA-mediated signaling associated with HPRT-deficiency, we examined the expression of a number of PDEs that are known to be highly expressed in the brain and regulate cAMP (PDE1C, PDE4B, PDE10A, PDE7B) in both control and HPRT-deficient MN9D cells [26], [27]. Figure 4 shows that only PDE10 expression is significantly increased in HPRT-deficient MN9D cells (Fig. 4A &4B). To investigate the contribution of increased PDE10 to the reduced cAMP/PKA-signaling associated with these HPRT-deficient cells, we examine substrate expression, including phospho-synapsin, in the presence of papaverine a PDE10 specific inhibitor [28], [29]. Papaverine significantly increased the level of PKA expression and phospho-PKA substrates expression, including phospho-synapsin, both before and after forskolin treatment (Figure 5A, B). In parallel, papaverine restored cAMP production accumulation in HPRT-deficient cells (data not shown). Increased phospho-PKA substrates were also seen in the presence of IBMX, a PDE non-specific inhibitor (data not shown), however not in presence other PDEs inhibitors such as Rolipram (PDE4, 30 µM), Vinpocetine (PDE1, 30 µM) and BRL50481 (PDE7, 50 µM) (See Figure S5). Although papaverine is used as PDE10 inhibitor, it is also known to have a number of non-specific effects [30]. Therefore, we used siRNA directed specifically to the mouse PDE10A gene to decrease PDE10A expression in HPRT-deficient MN9D cells. PDE10A protein expression was reduced in HPRT-deficient MN9D cells transfected with PDE10A siRNA compared to scramble (control) siRNA (Figure 5C, D). The decrease of PDE10A gene expression by siRNA-PDE10 in the HPRT-deficient cells significantly increased the expression of phospho-PKA substrates, including phospho-synapsin (Fig. 5E and 5F).

### Restoration of HPRT Expression and its Impact of Synapsin Expression and PKA-related Signaling

**Figure 6:**
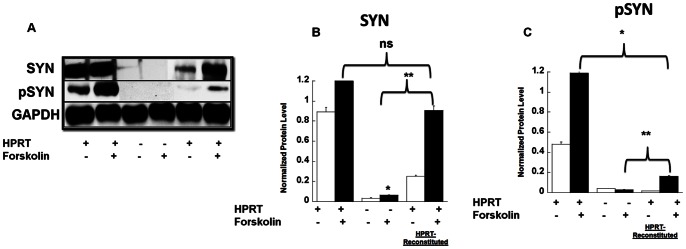
Effect of HPRT-rescue on cAMP/PKA signaling. Immuno-blot and densitometry quantification analysis of Syn 1and phospho-Syn I (**A, B & C**). Data show that the lower expression of Synapsin I protein in response to forskolin treatment in HPRT-deficient MN9D cells is abrogated in HPRT-reconstituted MN9D cells (**B**). p-Syn I (Ser9) expression in HPRT-reconstituted cells is partially restored (**C**). Error bars represent mean ± SEM of duplicate measurements of two independent experiments (n = 4). The asterisk (*) represents statistical significance (p<0.05) between forskolin treated cells control versus HPRT-deficient cells, the double asterisks (**), between forskolin-treated HPRT-deficient cells versus forskolin-treated HPRT-reconstituted cells (ANOVA and Tukey, post-hoc test).

To confirm the association between HPRT-deficiency and the decreased Synapsin I and PKA activity, we reconstituted HPRT expression and activity in HPRT negative MN9D cells. MN9D cells were transfected with lentivirus vectors encoding for GFP (transfection control) or the HPRT gene (see figure S6 in supplemental data). The HPRT-reconstituted MN9D mutant cells increased sensitivity to growth in culture medium containing 6-thio-guanine, (250 µM) unlike their GFP infected counterparts (data not shown). The HPRT-reconstituted MN9D cells displayed a noticeable increase in Syn I protein expression compared to HPRT-deficient MN9D cells (Fig. 6A–6B); however we observed only partial restoration of phospho-synapsin expression (15% of the control, Fig. 6C). Furthermore, the rescue of HPRT-activity did not fully restore the expression of phospho-PKA substrates or the increased expression of PDE10 in HPRT-deficient MN9D cell lines (data not shown).

## Discussion

We have previously shown that the dysregulation of miR-181a in HPRT-deficient cells affect the expression of key transcription factors that drive neuronal development [7]. The current study expands upon this previous investigation based on the premise that the actions of this microRNA extend to pathways beyond neurodevelopment to affect expression and signaling pathways of mature neurons. We identified that miRNA-181a may regulate the ubiquitously expressed transcription factor CREB, whose the role in neuronal pathway and function is widely documented [31], [32]. We demonstrate reduced expression of CREB in HPRT-deficient cells that correlated with reduced cAMP accumulation (Fig. 1). These data correlate with previous studies that have shown that HRPT-deficiency is accompanied by decreased activity of AC, which catalyzes the formation of cAMP [33]. A more recent study demonstrated the impaired expression and function of AC2 in HPRT-deficient rat B103 neuroblastoma [34]. Our data further support the role of dysfunctional cAMP-signaling in HPRT-deficiency.

As a transcription factor, CREB regulates the expression of a wide number of genes, such as the Synapsin gene which possesses a CRE consensus sequence in its promoter [35]. We demonstrate blunted expression of Synapsin I mRNA in HPRT-deficient MN9D cells (Fig. 2). Synapsin I, along with Synapsin II and Synapsin III are phosphoproteins that bind to the cytosolic surface of synaptic vesicles. Their role is to store and release neurotransmitters. Our current data suggest a role for Synapsin I in the etiology of HPRT-deficiency; its reduced expression may contribute to the alteration of the dopaminergic neurotransmitter system in LND [2], [36]. The reduction in agonist induced cAMP accumulation in HPRT-deficient cells led us to examine PKA-mediated signaling; since the cellular actions of cAMP are known to be mediated, at least in part, by PKA. In the brain, PKA phosphorylates many targets important for proper neuronal function [37]. Consequently, consistent with the reduction in cAMP production in HPRT-deficient cells, we found blunted PKA-mediated signaling in response to forskolin (Fig. 3). We report for the first time a global reduction of phospho-PKA-substrates including phospho-synapsin in HPRT-deficient MN9D cells both before and after agonist stimulation. We have demonstrated that the reduction in the expression of phospho-synapsin is also seen in HPRT-deficient human neuroblastoma cells (Fig. S4). Consistent with our findings is the observation of a global reduction of phospho-PKA-substrate expression in HPRT-deficient human induced pluripotent stem cells (Zhu and Friedmann, unpublished data). We also uncovered a decrease in the expression of many other PKA substrates in HPRT-deficient human and mouse neuroblastoma cell lines, including tyrosine hydroxylase (TH) (Ser40) and DARPP-32 (Thr34) (data not shown) that are also important effectors of neuronal function [28], [38], [39]. The reduced expression of these PKA dependent phospho-proteins, including synapsin, may have implications for LND neuropathophysiology.

Within the cell, the level of cAMP and thus PKA activity is mediated by a balance of formation by adenylyl cyclase, and degradation by PDEs [40]. From the list of potential target genes for miR-181A we found PDE10A to be potentially unregulated and therefore we further investigated the role of this PDE isoform in our cellular model (Table S3). While PDE3B and PDE5 are also among the potential targets of miR-181a, we did not examine their role in the cAMP/PKA dysregulation, principally because PDE3B expression in the brain is mostly known be associated with hypothalamic functions [41]–[43], while PDE5 controls cyclic GMP (cGMP) signaling. In the context of this study, and as PDE10 is known to hydrolyze both cAMP and cGMP, our data suggest a role for cGMP-dependent signaling in HPRT-deficiency. In fact, we have observed blunted production of cGMP along with dysregulated expression of protein kinase G (PKG) related signaling in HPRT-deficient MN9D cells upon exposure to nitric oxide (NO) donor Sodium nitroprusside (SNP) (Guibinga et al. unpublished data). We are currently investigating the significance of these findings in HPRT-deficiency and in relation, not only to PDEs dysregulation, but also uric acid, a known effector of NO production, which could indirectly influence cGMP/PKG signaling in HPRT-deficient cells.

We analyzed the expression of other PDEs known to hydrolyze cAMP, such as PDE4 and PDE7, but found these did not significantly change between control and HPRT-deficient cells (Fig. 4).We also examined the expression of PDE1C, a PDE1 isoform known to be expressed at higher level than PDE1A or PDE1B in the dopaminergic regions of substantia nigra [44]; like for PDE4 and PDE7, we did not observe a significant difference in PDE1C expression between control and HPRT-deficient cell lines (Fig. 4). Conversely, we found that HPRT-deficient cells had increased expression of PDE10A, suggesting enhanced PDE10 activity. We have also found an increase in PDE10 mRNA expression in fibroblasts cells derived from LND patients compared to control (Figure S7). Mindful that fibroblast and neuronal cells have a different genomic make up, in absence of brain tissues derived from LND patients, fibroblast cells have often validated dysregulated expression of genes or microRNAs relevant to brain functions ([3], [7]. Of interest, PDE10 expression is highly enriched in the striatum [45], part of the forebrain known to be affected in LND. We have shown an increase of PDE10 transcripts in striatal tissue derived from HPRT knockout (HPRTKO) relative to wild-type (Guibinga, unpublished data). All together, these results support a role for PDE10A in the etiology of LND. These data also suggest caution as the full dynamic of PDEs expression in LND brains and HPRTKO mouse striatum remain to be determined.

Papaverine, a PDE10 specific inhibitor, and siRNA targeted to PDE10A reversed the decrease in expression of phospho-PKA-substrates, including phospho-synapsin, in HPRT-deficient MN9D cells. Together these data suggest a key role for PDE10A in the dysfunctional cAMP signaling in these HPRT-deficient cells. Inhibitors of PDE10 have been shown to increase CREB phosphorylation in both mouse striatum in vivo and rat striatal neurons in vivo [46], [47]. Moreover, new generation of PDE10 inhibitors more potent and more specific than papaverine are currently under investigation as novel antipsychotic agents [24], [48]. Pending the full characterization of PDEs expression and activity profile in the basal ganglia of LND patients, these new generations of PDE10 inhibitors could potentially be evaluated as therapeutic agents for LND; a disease for which there are still limited therapeutic options. In the meantime the unraveling of cyclic nucleotide regulation dynamic in the striatum of HPRTKO mouse through pharmacological manipulation could provide initial cues on the potential applicability of PDE-based therapy for LND.

Until now, only few studies have examined the role that cyclic nucleotides play in the etiology of HPRT-deficiency. Two of these investigations have documented significant changes in cAMP level and ACs in cell membranes from HPRT-deficient cell lines [33], [34]. Although, these two studies have been immensely informative in linking HPRT-deficiency to cyclic AMP related functions, their data did not correlate these cAMP-alterations to signaling pathways known to be activated by cAMP, nor did they demonstrate their relevance to neuronal functioning. Our investigation investigates for the first time the role of PDEs in HPRT-deficient cells; in addition, our study provides a novel angle on the cyclic nucleotides regulation in HPRT-deficiency and suggests the impact they may have of neuronal function.

To investigate further how HPRT-deficiency affects cAMP/PKA signaling, we restored HPRT expression and activity; our data show the restoration of Syn I expression in HPRT-reconstituted cells (Fig. 6A & 6B). In contrast, we only partially reversed the reduced level of phospho-synapsin I in HPRT-reconstituted MN9D cells. These data show that the reduced expression of synapsin mRNA and protein, as shown in figures 2, 3 and 5, is only partially responsible for the reduced phospho-synapsin. We propose that the derangements in purine metabolism caused by HPRT-deficiency can lead to accumulation of metabolites that affect PKA-signaling, and those are not necessarily reversible by restoration of HPRT expression and activity. Our findings are consistent with previous research carried out in HPRT-deficient PC12 cell culture; where HPRT-deficiency was shown to affect dopamine content in these cells, while the rescue of HPRT failed to restore the dopamine level to normal [49].

**Figure 7:**
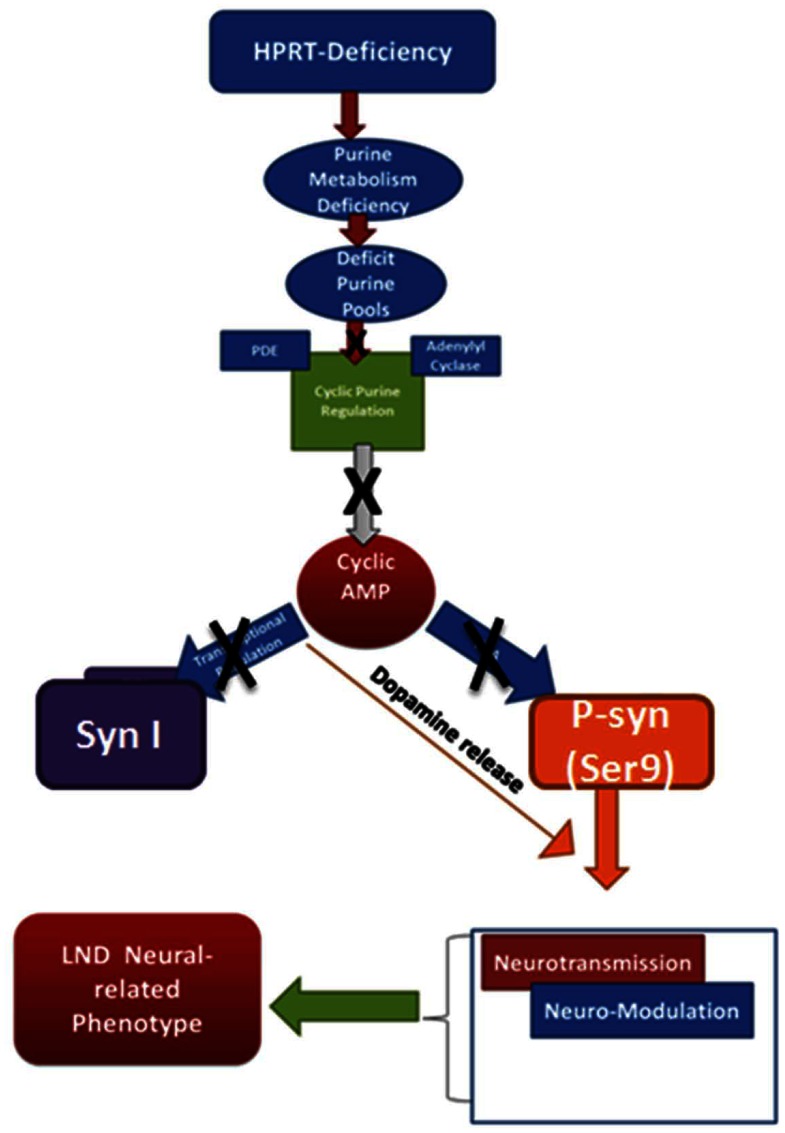
Schematic of the possible mechanisms by which HPRT-deficiency via blunting of cAMP/PKA signaling leads to the neural-related LND phenotype. HPRT-deficiency is primarily a purine metabolism deficiency that affects the cellular purine pools. The deficit in purine pools causes changes in expression and activity of PDE and/or AC reduces cAMP production. We propose that the cAMP deficit in HPRT-deficient causes a reduction in CREB and thereby expression of neural genes such as Synapsin I. The decrease in PKA activity blunts phosphorylation of substrates, such as p-Syn I (Ser9), which is known to affect neurotransmitter release. We conclude HPRT-deficiency via inhibiting cAMP/PKA signaling could affect neurotransmission and neuro-modulation that underlie the neurological phenotype in LND.

All together our data suggests a novel mechanism by which in HPRT-deficiency contributes to neural dysfunction. We propose blunted CREB-mediated transcriptional and cAMP/PKA signaling, possibly due in part to increased PDE10A, provides a rationale and coherent framework of a possible mechanism by which HPRT-deficiency affects the neuro-pathogenesis of LND. As depicted in Figure 7, the purine metabolism alterations caused by HPRT-deficiency engenders deficits in cyclic AMP regulation which in turn affect PKA signaling, among other phospho-synapsin expression. Blunted phospho-synapsin expression likely impacts dopamine release, thus neurotransmission and neuro-modulation thereby causing the neurological phenotype in LND.

## Supporting Information

Figure S1 **HPRT-knockdown in HEK293 cells.** Western blot analysis of HEK293 cells infected with Lentivector-sh2hprt expressing the small hairpin targeted to HPRT (SH2-HPRT, right lane) or with control vector Lentivector-shlux targeted against luciferase (WT-LUX, left lane). Figure shows a significant reduction of HPRT protein in Lentivector-sh2prt-transduced cells.
(TIF)

Figure S2 **Reduced agonist induced cAMP accumulation in HPRT-deficient cells.** **(A)** HPRT-deficient human 293 (SH2-HPRT) cell lines and their equivalent control cell lines (Sh-LUX) were stimulated with DMSO (CTL) and forskolin 50 µM 15 min. cyclic AMP level was evaluated as described in material and methods. The data are expressed as level of cAMP normalized to protein content. Error bars represent mean ± SEM of duplicate measurements of two independent experiments (n = 4). The asterisks (*p<0.05) represent statistical significance between forskolin treated cells (t-test).
(TIF)

Figure S3 **HPRT-deficiency blunts cyclic AMP-dependent protein kinase (PKA) activity.** Figure shows decreased PKA activity in HPRT deficient MN9D cells. The data are expressed as normalized level of PKA activity relative to total protein content. Error bars represent mean ± SEM of duplicate measurements of two independent experiments (n = 4). The asterisks represent statistical significance between forskolin treated cells (*p<0.05, t-test).
(TIF)

Figure S4 **Reduced phospho-synapsin in human HPRT-deficient SH-SY5Y cells.** **(A & B),** immuno-blot and quantification analysis of p-Syn (Ser9), data show that the lower expression of syn I and p-Syn (Ser9) in response to forskolin treatment in HPRT-deficient SH-SY5Y cells. Error bars represent mean ± SEM of duplicate measurements of two independent experiments (n = 4). The asterisk (*) represent statistical significance between forskolin treated cells (*p<0.05, t-test).
(TIF)

Figure S5 **The PDE1, PDE4 and PDE7 inhibitors vinpocetine, rolipram and BRL5081, respectively do not improve phospho-synapsin p-Syn (Ser9) expression in HPRT-deficient MN9D cells.** (A, B & C) immuno-blot of control and HPRT-deficient MN9D cells after pre-treated with the indicated PDEs inhibitors before forskolin treatment (see methods).
(TIF)

Figure S6 **HPRT-rescue in HPRT-deficient MN9D cells. (A)** HPRT mRNA in MN9D deficient cells infected with lentivector encoding the GFP gene (GFP) or the HPRT gene (HPRT). The data show a significant increase of HPRT gene expression over the GFP expressing cells (41 fold). Error bars represent mean ± SEM of duplicate measurements (n = 2). The asterisk (*) represents statistical significance (p<0.05, t-test) between GFP-infected cells and HPRT-infected cells. **(B &C)**, reconstitution of HPRT expression in HPRT-deficient MN9D cells. Immuno-blot and quantification analysis of HPRT protein expression in control MN9D cells (1), HPRT-deficient MN9D cells (2), HPRT-lentivirus infected HPRT-deficient MN9D cells (3), and GFP-lentivirus infected HPRT-deficient MN9D cells (4). Error bars represent mean ± SEM of triplicate measurements (n = 3). The asterisk (*) represents the statistical significance (p<0.05) between the control and HPRT-deficient MND9 cells; while the double asterisks **represent statistical significance between GFP and HPRT-infected cell cells (p<0.05, t-test).
(TIF)

Figure S7 **Gene expression profile of PDE10A in fibroblasts cells from normal (CTL) mildly (LNV) and severely (LND) affected HPRT-deficient patients.** (A) ΔCT value of each category of patients, showing that LND subjects have significantly lower ΔCT than control (CTL); leading to significantly higher fold change in PDE10 mRNA level (B). (* p<0.05, ANOVA).
(TIF)

Table S1 **List of primers.**
(TIF)

Table S2 **Functional annotation clustering pertaining to purine pathways.** The table includes GO terms related to “purine metabolism” derived from miR-181a potential target genes and selected from target-combo and targetScan database. GO terms were extracted using DAVID as previously described (Guibinga et al. 2012). Highlighted is the GO term “purine nucleotide metabolic process”.
(TIF)

Table S3 **List of potential miR-181a target genes derived from GO term related to “purine nucleotide metabolic process”.** The table includes several genes known to regulate cAMP/PKA, such as PDE10.
(TIF)

## References

[1] Gedye A (1992) Serotonin-gaba Treatment is Hypothesized for Self-injury in Lesch-Nyhan Syndrome. Medical Hupotheses 38: 325–328. 10.1016/0306-9877(92)90026-9 1491633

[2] Jinnah HA, Friedmann T (2000) Lesch-Nyhan Disease and its variants. In: Scriver C, Beaudet, AL, Sly WS, Valle, D., editor. The Metabolic and Molecular bases of inherited disease. 8 ed ed: McGraw-Hill. 2537–2570.

[3] Ceballos-Picot I, Mockel L, Potier MC, Dauphinot L, Shirley TL, et al (2009) Hypoxanthine-guanine phosphoribosyl transferase regulates early developmental programming of dopamine neurons: implications for Lesch-Nyhan disease pathogenesis. Hum Mol Genet 18: 2317–2327. 19342420 10.1093/hmg/ddp164 PMC2694685

[4] Guibinga GH, Hsu S, Friedmann T (2010) Deficiency of the housekeeping gene hypoxanthine-guanine phosphoribosyltransferase (HPRT) dysregulates neurogenesis. Mol Ther 18: 54–62. 19672249 10.1038/mt.2009.178 PMC2839227

[5] Cristini S, Navone S, Canzi L, Acerbi F, Ciusani E, et al (2010) Human neural stem cells: a model system for the study of Lesch-Nyhan disease neurological aspects. Hum Mol Genet 19: 1939–1950. 20159777 10.1093/hmg/ddq072

[6] Kang TH, Guibinga GH, Jinnah HA, Friedmann T (2011) HPRT deficiency coordinately dysregulates canonical Wnt and presenilin-1 signaling: a neuro-developmental regulatory role for a housekeeping gene? PLoS One 6: e16572. 21305049 10.1371/journal.pone.0016572 PMC3030599

[7] Guibinga GH, Hrustanovic G, Bouic K, Jinnah HA, Friedmann T (2012) MicroRNA-mediated dysregulation of neural developmental genes in HPRT deficiency: clues for Lesch-Nyhan disease? Hum Mol Genet 21: 609–622. 22042773 10.1093/hmg/ddr495 PMC3259014

[8] Lewers JC, Ceballos-Picot I, Shirley TL, Mockel L, Egami K, et al (2008) Consequences of impaired purine recycling in dopaminergic neurons. Neuroscience 152: 761–772. 18313225 10.1016/j.neuroscience.2007.10.065 PMC3498629

[9] Guibinga GH, Friedmann T (2005) Baculovirus GP64-pseudotyped HIV-based lentivirus vectors are stabilized against complement inactivation by codisplay of decay accelerating factor (DAF) or of a GP64-DAF fusion protein. Mol Ther 11: 645–651. 15771967 10.1016/j.ymthe.2004.12.002

[10] Mastrangelo L, Kim JE, Miyanohara A, Kang TH, Friedmann T (2012) Purinergic signaling in human pluripotent stem cells is regulated by the housekeeping gene encoding hypoxanthine guanine phosphoribosyltransferase. Proc Natl Acad Sci U S A 109: 3377–3382. 22331909 10.1073/pnas.1118067109 PMC3295269

[11] Murray F, Patel HH, Suda RY, Zhang S, Thistlethwaite PA, et al (2007) Expression and activity of cAMP phosphodiesterase isoforms in pulmonary artery smooth muscle cells from patients with pulmonary hypertension: role for PDE1. Am J Physiol Lung Cell Mol Physiol 292: L294–303. 16980375 10.1152/ajplung.00190.2006

[12] Ostrom RS, Violin JD, Coleman S, Insel PA (2000) Selective enhancement of beta-adrenergic receptor signaling by overexpression of adenylyl cyclase type 6: colocalization of receptor and adenylyl cyclase in caveolae of cardiac myocytes. Mol Pharmacol 57: 1075–1079. 10779394

[13] Naldini L, Blömer U, Gallay P, Ory D, Mulligan R, et al (1996) In vivo gene delivery and stable transduction of nondividing cells by a lentiviral vector. Science 272: 263–267. 8602510 10.1126/science.272.5259.263

[14] Huang da W, Sherman BT, Lempicki RA (2009) Systematic and integrative analysis of large gene lists using DAVID bioinformatics resources. Nat Protoc 4: 44–57. 19131956 10.1038/nprot.2008.211

[15] Huang da W, Sherman BT, Lempicki RA (2009) Bioinformatics enrichment tools: paths toward the comprehensive functional analysis of large gene lists. Nucleic Acids Res 37: 1–13. 19033363 10.1093/nar/gkn923 PMC2615629

[16] Huang da W, Sherman BT, Zheng X, Yang J, Imamichi T, et al.. (2009) Extracting biological meaning from large gene lists with DAVID. Curr Protoc Bioinformatics Chapter 13: Unit 13 11. 10.1002/0471250953.bi1311s27 19728287

[17] Zhou Z, Kim J, Insolera R, Peng X, Fink DJ, et al (2011) Rho GTPase regulation of alpha-synuclein and VMAT2: implications for pathogenesis of Parkinson’s disease. Mol Cell Neurosci 48: 29–37. 21699982 10.1016/j.mcn.2011.06.002 PMC3163163

[18] Park B, Oh CK, Choi WS, Chung IK, Youdim MB, et al (2011) Microarray expression profiling in 6-hydroxydopamine-induced dopaminergic neuronal cell death. J Neural Transm 118: 1585–1598. 21904894 10.1007/s00702-011-0710-x

[19] Mayr B, Montminy M (2001) Transcriptional regulation by the phosphorylation-dependent factor CREB. Nat Rev Mol Cell Biol 2: 599–609. 11483993 10.1038/35085068

[20] Kim SS, Moon KR, Choi HJ (2011) Interference of alpha-synuclein with cAMP/PKA-dependent CREB signaling for tyrosine hydroxylase gene expression in SK-N-BE(2)C cells. Arch Pharm Res 34: 837–845. 21656370 10.1007/s12272-011-0518-0

[21] Beninger RJ, Miller R (1998) Dopamine D1-like receptors and reward-related incentive learning. Neurosci Biobehav Rev 22: 335–345. 9579323 10.1016/s0149-7634(97)00019-5

[22] Borgkvist A, Fisone G (2007) Psychoactive drugs and regulation of the cAMP/PKA/DARPP-32 cascade in striatal medium spiny neurons. Neurosci Biobehav Rev 31: 79–88. 16730373 10.1016/j.neubiorev.2006.03.003

[23] Evans GJ, Morgan A (2003) Regulation of the exocytotic machinery by cAMP-dependent protein kinase: implications for presynaptic plasticity. Biochem Soc Trans 31: 824–827. 12887314 10.1042/bst0310824

[24] Menniti FS, Chappie TA, Humphrey JM, Schmidt CJ (2007) Phosphodiesterase 10A inhibitors: a novel approach to the treatment of the symptoms of schizophrenia. Curr Opin Investig Drugs 8: 54–59. 17263185

[25] Hebb AL, Robertson HA, Denovan-Wright EM (2008) Phosphodiesterase 10A inhibition is associated with locomotor and cognitive deficits and increased anxiety in mice. Eur Neuropsychopharmacol 18: 339–363. 17913473 10.1016/j.euroneuro.2007.08.002

[26] Kelly MP, Brandon NJ (2009) Differential function of phosphodiesterase families in the brain: gaining insights through the use of genetically modified animals. Prog Brain Res 179: 67–73. 20302819 10.1016/S0079-6123(09)17908-6

[27] Xu Y, Zhang HT, O’Donnell JM (2011) Phosphodiesterases in the central nervous system: implications in mood and cognitive disorders. Handb Exp Pharmacol: 447–485. 10.1007/978-3-642-17969-3_19 21695652

[28] Nishi A, Kuroiwa M, Miller DB, O’Callaghan JP, Bateup HS, et al (2008) Distinct roles of PDE4 and PDE10A in the regulation of cAMP/PKA signaling in the striatum. J Neurosci 28: 10460–10471. 18923023 10.1523/JNEUROSCI.2518-08.2008 PMC2814340

[29] Spiwoks-Becker I, Wolloscheck T, Rickes O, Kelleher DK, Rohleder N, et al (2011) Phosphodiesterase 10A in the rat pineal gland: localization, daily and seasonal regulation of expression and influence on signal transduction. Neuroendocrinology 94: 113–123. 21474921 10.1159/000327138

[30] Truss MC, Uckert S, Stief CG, Forssmann WG, Jonas U (1996) Cyclic nucleotide phosphodiesterase (PDE) isoenzymes in the human detrusor smooth muscle. II. Effect of various PDE inhibitors on smooth muscle tone and cyclic nucleotide levels in vitro. Urol Res 24: 129–134. 8839479 10.1007/BF00304075

[31] Dworkin S, Mantamadiotis T (2010) Targeting CREB signalling in neurogenesis. Expert Opin Ther Targets 14: 869–879. 20569094 10.1517/14728222.2010.501332

[32] Merz K, Herold S, Lie DC (2011) CREB in adult neurogenesis–master and partner in the development of adult-born neurons? Eur J Neurosci 33: 1078–1086. 21395851 10.1111/j.1460-9568.2011.07606.x

[33] Pinto CS, Seifert R (2006) Decreased GTP-stimulated adenylyl cyclase activity in HPRT-deficient human and mouse fibroblast and rat B103 neuroblastoma cell membranes. J Neurochem 96: 454–459. 16336632 10.1111/j.1471-4159.2005.03570.x

[34] Kinast L, von der Ohe J, Burhenne H, Seifert R (2012) Impairment of adenylyl cyclase 2 function and expression in hypoxanthine phosphoribosyltransferase-deficient rat B103 neuroblastoma cells as model for Lesch-Nyhan disease: BODIPY-forskolin as pharmacological tool. Naunyn Schmiedebergs Arch Pharmacol 385: 671–683. 22552731 10.1007/s00210-012-0759-6

[35] Hoesche C, Bartsch P, Kilimann MW (1995) The CRE consensus sequence in the synapsin I gene promoter region confers constitutive activation but no regulation by cAMP in neuroblastoma cells. Biochim Biophys Acta 1261: 249–256. 7711068 10.1016/0167-4781(95)00014-8

[36] Jinnah HA, Langlais PJ, Friedmann T (1992) Functional analysis of brain dopamine systems in a genetic mouse model of Lesch-Nyhan syndrome. JPharmacolExpTher 263: 596–607. 1432691

[37] Browning MD, Huganir R, Greengard P (1985) Protein phosphorylation and neuronal function. J Neurochem 45: 11–23. 2582086 10.1111/j.1471-4159.1985.tb05468.x

[38] Svenningsson P, Nishi A, Fisone G, Girault JA, Nairn AC, et al (2004) DARPP-32: an integrator of neurotransmission. Annu Rev Pharmacol Toxicol 44: 269–296. 14744247 10.1146/annurev.pharmtox.44.101802.121415

[39] Hemmings HC Jr, Nairn AC, McGuinness TL, Huganir RL, Greengard P (1989) Role of protein phosphorylation in neuronal signal transduction. FASEB J 3: 1583–1592. 2493406 10.1096/fasebj.3.5.2493406

[40] Baillie GS, Scott JD, Houslay MD (2005) Compartmentalisation of phosphodiesterases and protein kinase A: opposites attract. FEBS Lett 579: 3264–3270. 15943971 10.1016/j.febslet.2005.03.089

[41] Sahu A (2011) Intracellular leptin-signaling pathways in hypothalamic neurons: the emerging role of phosphatidylinositol-3 kinase-phosphodiesterase-3B-cAMP pathway. Neuroendocrinology 93: 201–210. 21464566 10.1159/000326785 PMC3130491

[42] Sahu A (2010) A role of phosphodiesterase-3B pathway in mediating leptin action on proopiomelanocortin and neurotensin neurons in the hypothalamus. Neurosci Lett 479: 18–21. 20471454 10.1016/j.neulet.2010.05.018 PMC2893383

[43] Sahu M, Litvin DG, Sahu A (2011) Phosphodiesterase-3B is expressed in proopiomelanocortin and neuropeptide Y neurons in the mouse hypothalamus. Neurosci Lett 505: 93–97. 22001576 10.1016/j.neulet.2011.09.068 PMC3221597

[44] Lakics V, Karran EH, Boess FG (2010) Quantitative comparison of phosphodiesterase mRNA distribution in human brain and peripheral tissues. Neuropharmacology 59: 367–374. 20493887 10.1016/j.neuropharm.2010.05.004

[45] Seeger TF, Bartlett B, Coskran TM, Culp JS, James LC, et al (2003) Immunohistochemical localization of PDE10A in the rat brain. Brain Res 985: 113–126. 12967715 10.1016/s0006-8993(03)02754-9

[46] Siuciak JA, Chapin DS, Harms JF, Lebel LA, McCarthy SA, et al (2006) Inhibition of the striatum-enriched phosphodiesterase PDE10A: a novel approach to the treatment of psychosis. Neuropharmacology 51: 386–396. 16780899 10.1016/j.neuropharm.2006.04.013

[47] Xie Z, Adamowicz WO, Eldred WD, Jakowski AB, Kleiman RJ, et al (2006) Cellular and subcellular localization of PDE10A, a striatum-enriched phosphodiesterase. Neuroscience 139: 597–607. 16483723 10.1016/j.neuroscience.2005.12.042 PMC1464838

[48] Yang SW, Smotryski J, McElroy WT, Tan Z, Ho G, et al (2012) Discovery of orally active pyrazoloquinolines as potent PDE10 inhibitors for the management of schizophrenia. Bioorg Med Chem Lett 22: 235–239. 22142545 10.1016/j.bmcl.2011.11.023

[49] Bitler CM, Howard BD (1986) Dopamine metabolism in hypoxanthine-guanine phosphoribosyltransferase-deficient variants of PC12 cells. Journal of Neurochemistry 47: 107–112. 3519867 10.1111/j.1471-4159.1986.tb02837.x

