# Supplementary figures and images for: HPRT-Deficiency Dysregulates cAMP-PKA Signaling and Phosphodiesterase 10A Expression: Mechanistic Insight and Potential Target for Lesch-Nyhan Disease?
Source: PLoS One. 2013 May 14;8(5):e63333. doi: 10.1371/journal.pone.0063333 (PMC3653951; doi:10.1371/journal.pone.0063333)

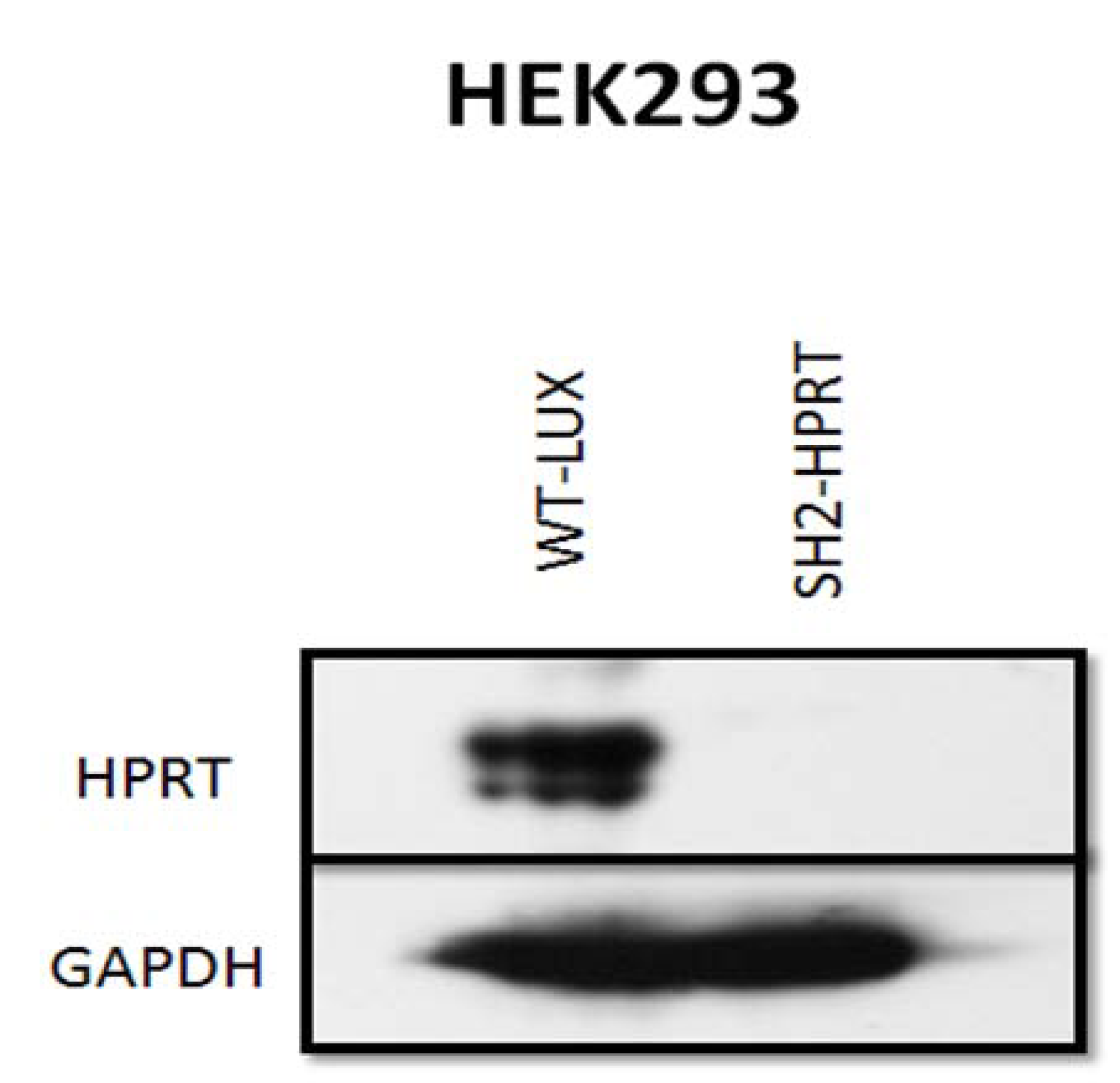

Supplement: Figure S1 — HPRT-knockdown in HEK293 cells. Western blot analysis of HEK293 cells infected with Lentivector-sh2hprt expressing the small hairpin targeted to HPRT (SH2-HPRT, right lane) or with control vector Lentivector-shlux targeted against luciferase (WT-LUX, left lane). Figure shows a significant reduction of HPRT protein in Lentivector-sh2prt-transduced cells. (TIF) [file pone.0063333.s001.tif]

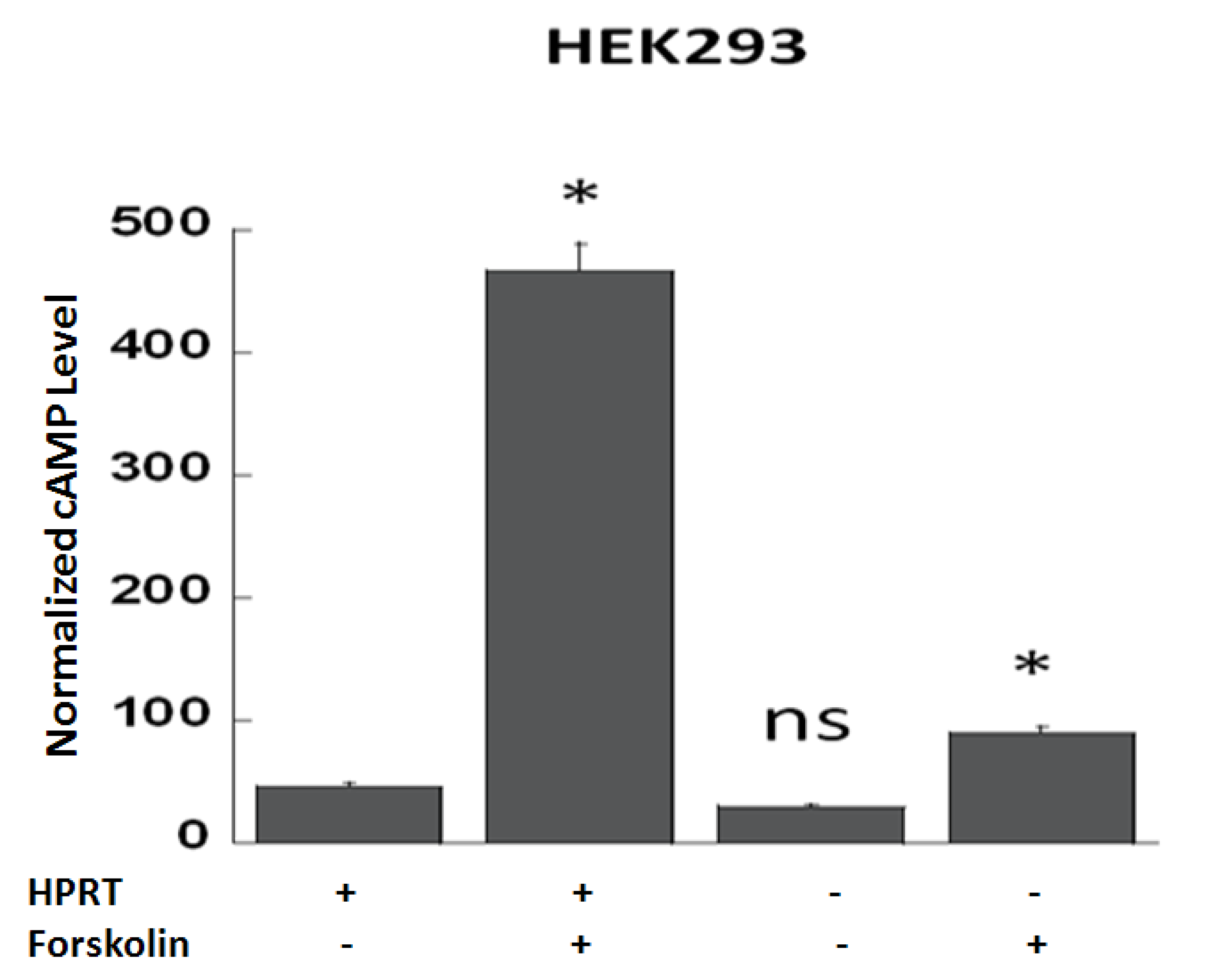

Supplement: Figure S2 — Reduced agonist induced cAMP accumulation in HPRT-deficient cells. (A) HPRT-deficient human 293 (SH2-HPRT) cell lines and their equivalent control cell lines (Sh-LUX) were stimulated with DMSO (CTL) and forskolin 50 µM 15 min. cyclic AMP level was evaluated as described in material and methods. The data are expressed as level of cAMP normalized to protein content. Error bars represent mean ± SEM of duplicate measurements of two independent experiments (n = 4). The asterisks (*p<0.05) represent statistical significance between forskolin treated cells (t-test). (TIF) [file pone.0063333.s002.tif]

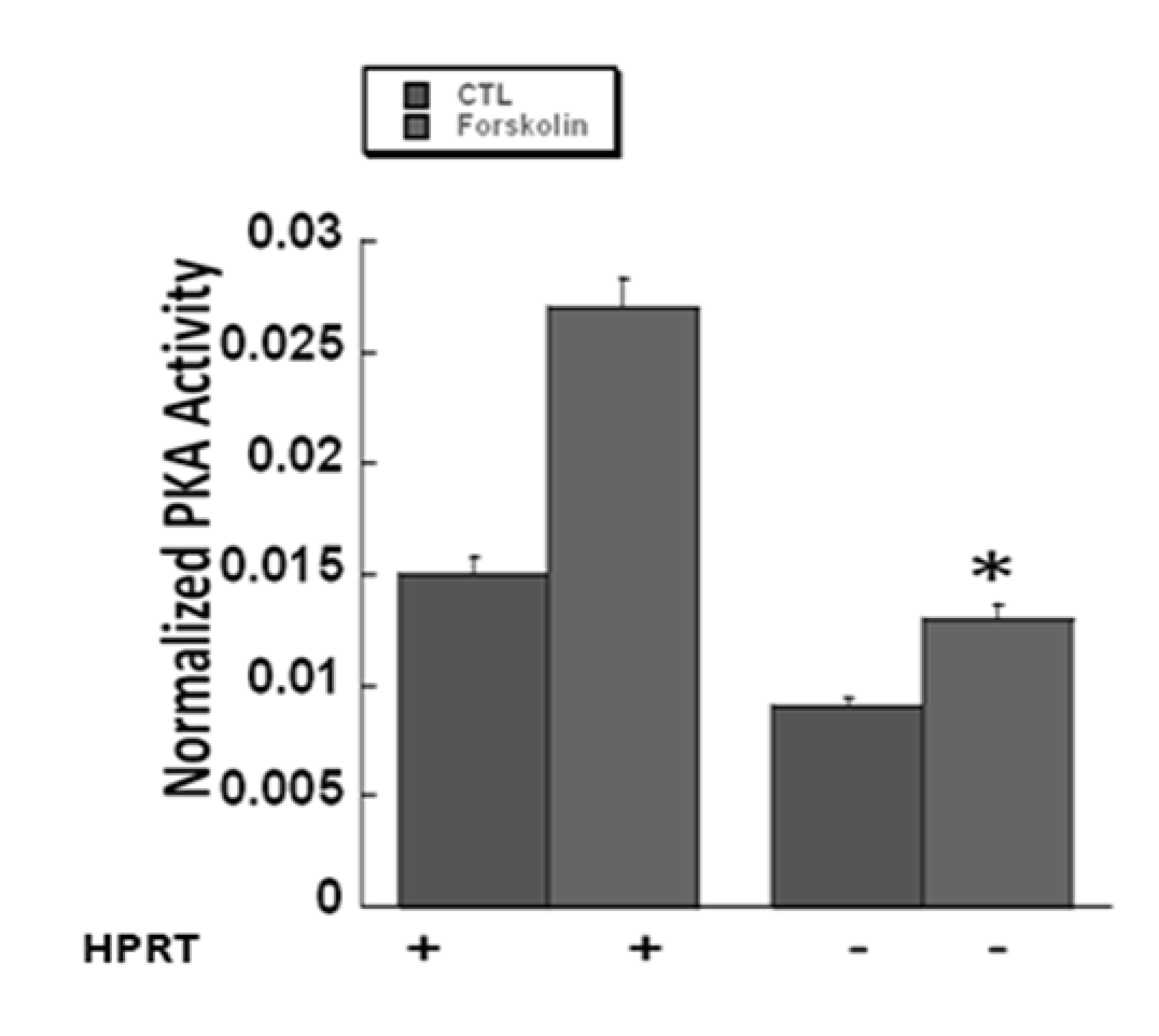

Supplement: Figure S3 — HPRT-deficiency blunts cyclic AMP-dependent protein kinase (PKA) activity. Figure shows decreased PKA activity in HPRT deficient MN9D cells. The data are expressed as normalized level of PKA activity relative to total protein content. Error bars represent mean ± SEM of duplicate measurements of two independent experiments (n = 4). The asterisks represent statistical significance between forskolin treated cells (*p<0.05, t-test). (TIF) [file pone.0063333.s003.tif]

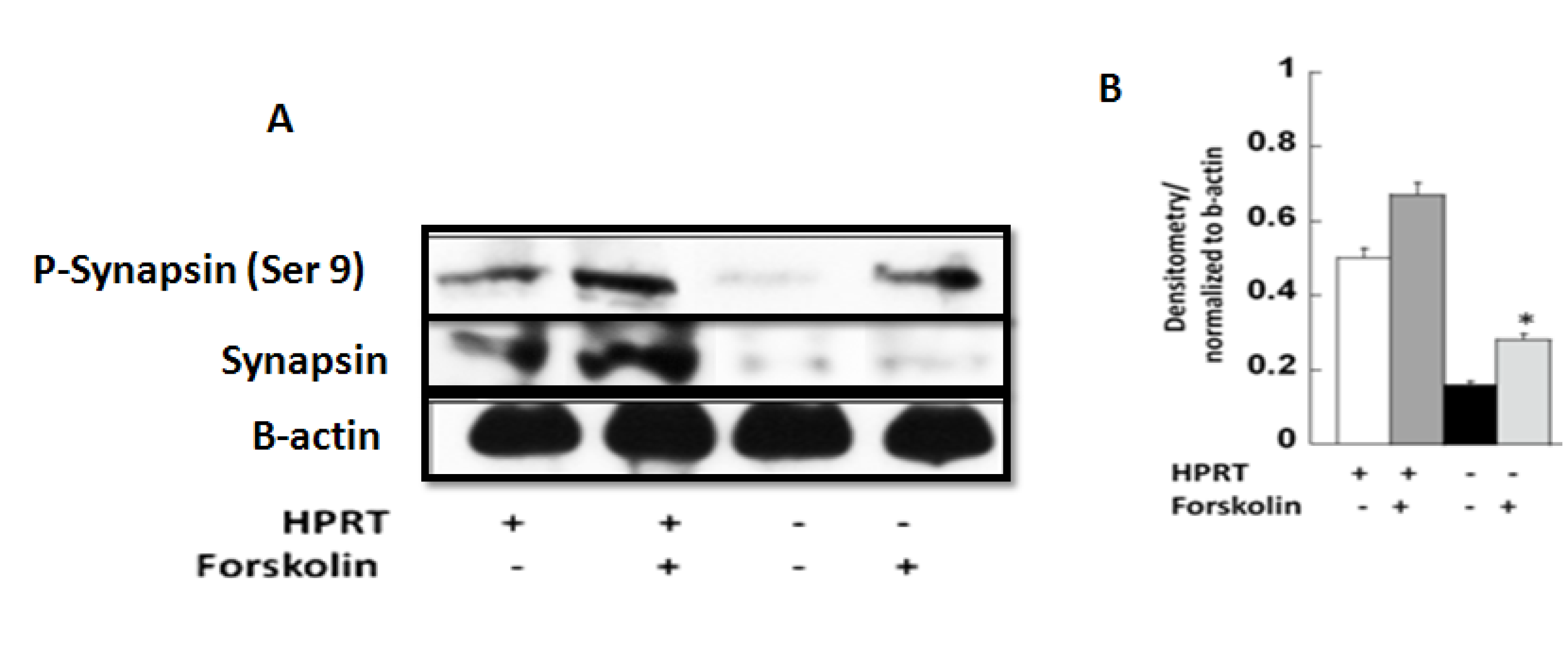

Supplement: Figure S4 — Reduced phospho-synapsin in human HPRT-deficient SH-SY5Y cells. (A & B), immuno-blot and quantification analysis of p-Syn (Ser9), data show that the lower expression of syn I and p-Syn (Ser9) in response to forskolin treatment in HPRT-deficient SH-SY5Y cells. Error bars represent mean ± SEM of duplicate measurements of two independent experiments (n = 4). The asterisk (*) represent statistical significance between forskolin treated cells (*p<0.05, t-test). (TIF) [file pone.0063333.s004.tif]

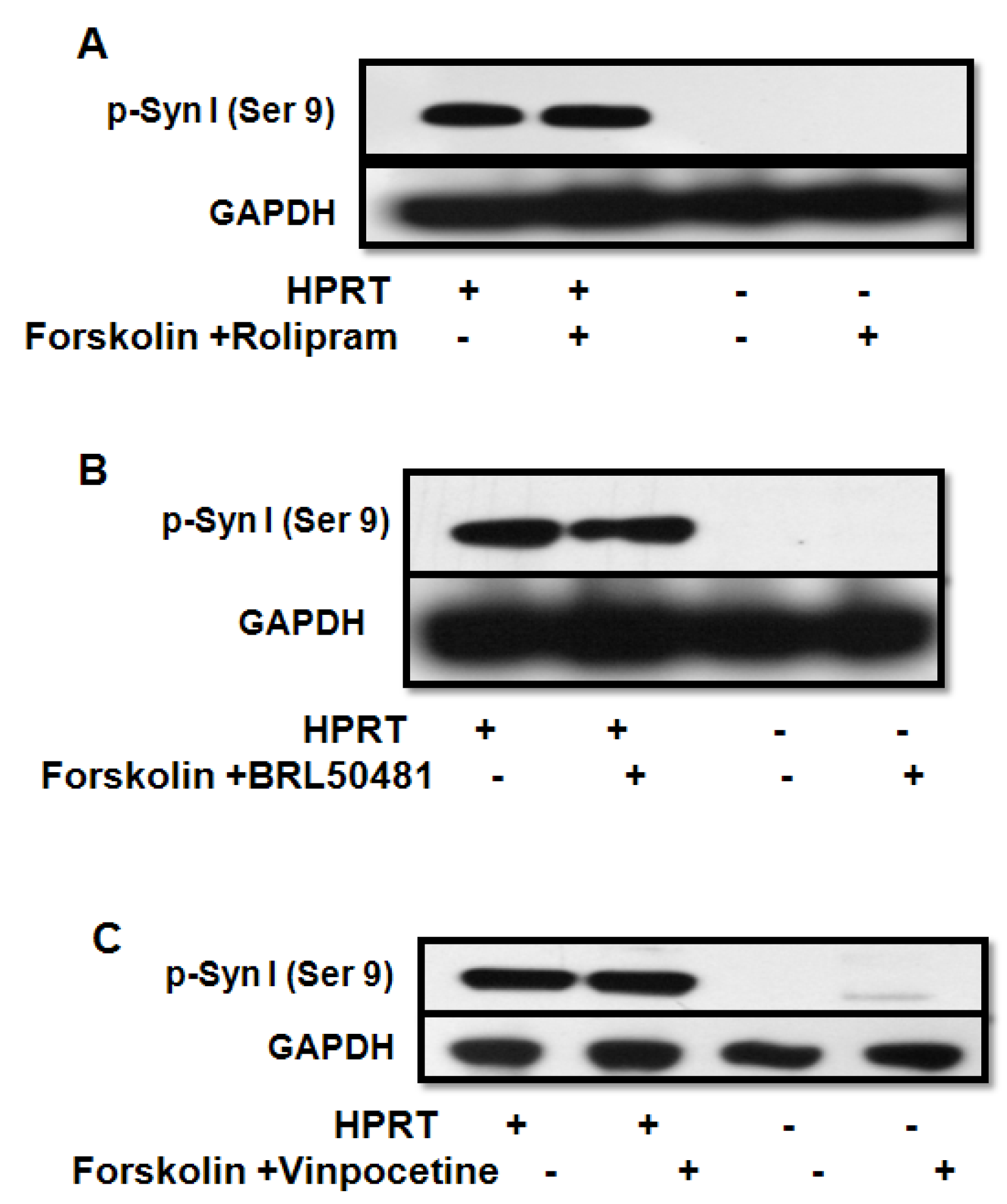

Supplement: Figure S5 — The PDE1, PDE4 and PDE7 inhibitors vinpocetine, rolipram and BRL5081, respectively do not improve phospho-synapsin p-Syn (Ser9) expression in HPRT-deficient MN9D cells. (A, B & C) immuno-blot of control and HPRT-deficient MN9D cells after pre-treated with the indicated PDEs inhibitors before forskolin treatment (see methods). (TIF) [file pone.0063333.s005.tif]

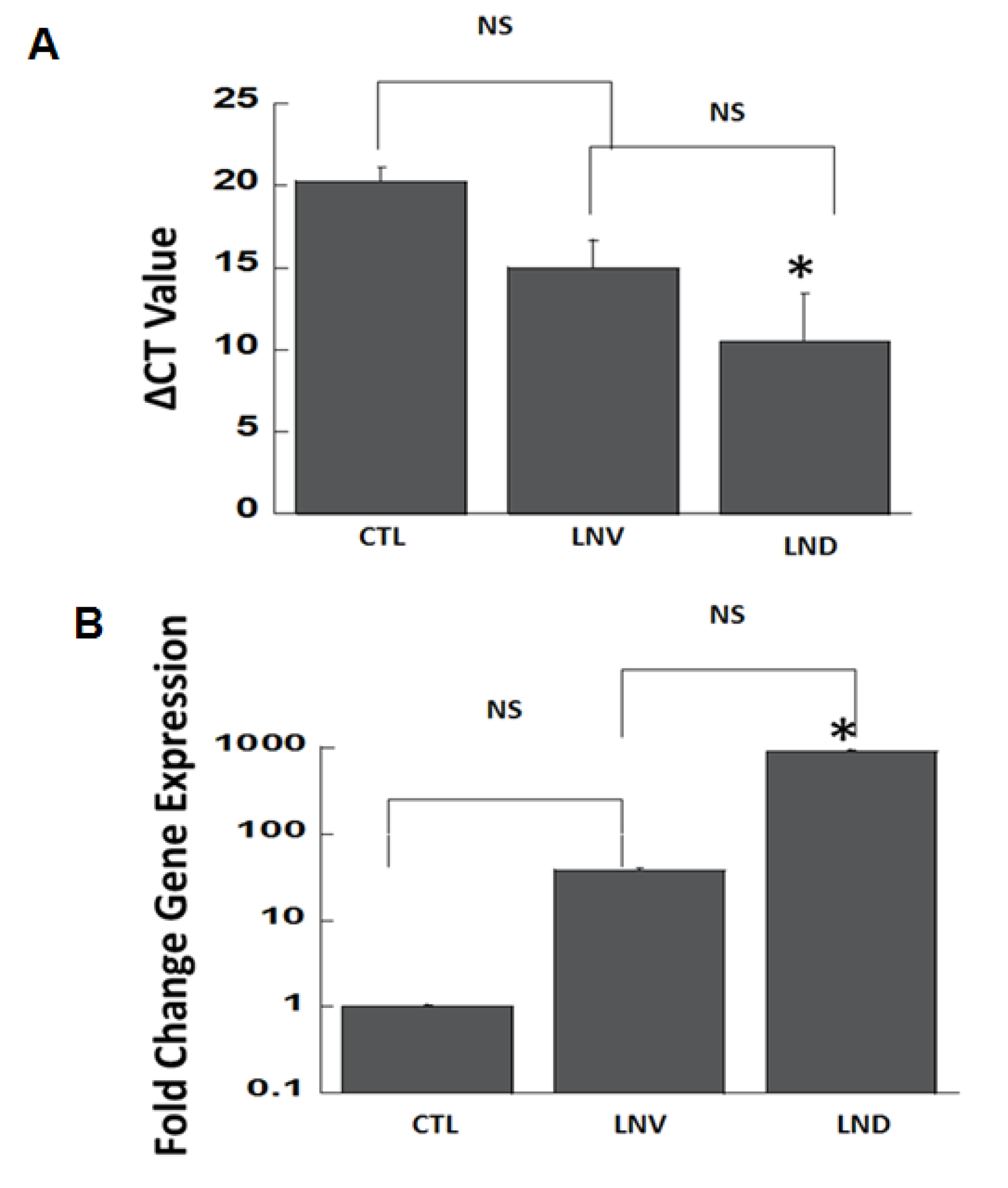

Supplement: Figure S6 — HPRT-rescue in HPRT-deficient MN9D cells. (A) HPRT mRNA in MN9D deficient cells infected with lentivector encoding the GFP gene (GFP) or the HPRT gene (HPRT). The data show a significant increase of HPRT gene expression over the GFP expressing cells (41 fold). Error bars represent mean ± SEM of duplicate measurements (n = 2). The asterisk (*) represents statistical significance (p<0.05, t-test) between GFP-infected cells and HPRT-infected cells. (B &C), reconstitution of HPRT expression in HPRT-deficient MN9D cells. Immuno-blot and quantification analysis of HPRT protein expression in control MN9D cells (1), HPRT-deficient MN9D cells (2), HPRT-lentivirus infected HPRT-deficient MN9D cells (3), and GFP-lentivirus infected HPRT-deficient MN9D cells (4). Error bars represent mean ± SEM of triplicate measurements (n = 3). The asterisk (*) represents the statistical significance (p<0.05) between the control and HPRT-deficient MND9 cells; while the double asterisks **represent statistical significance between GFP and HPRT-infected cell cells (p<0.05, t-test). (TIF) [file pone.0063333.s006.tif]

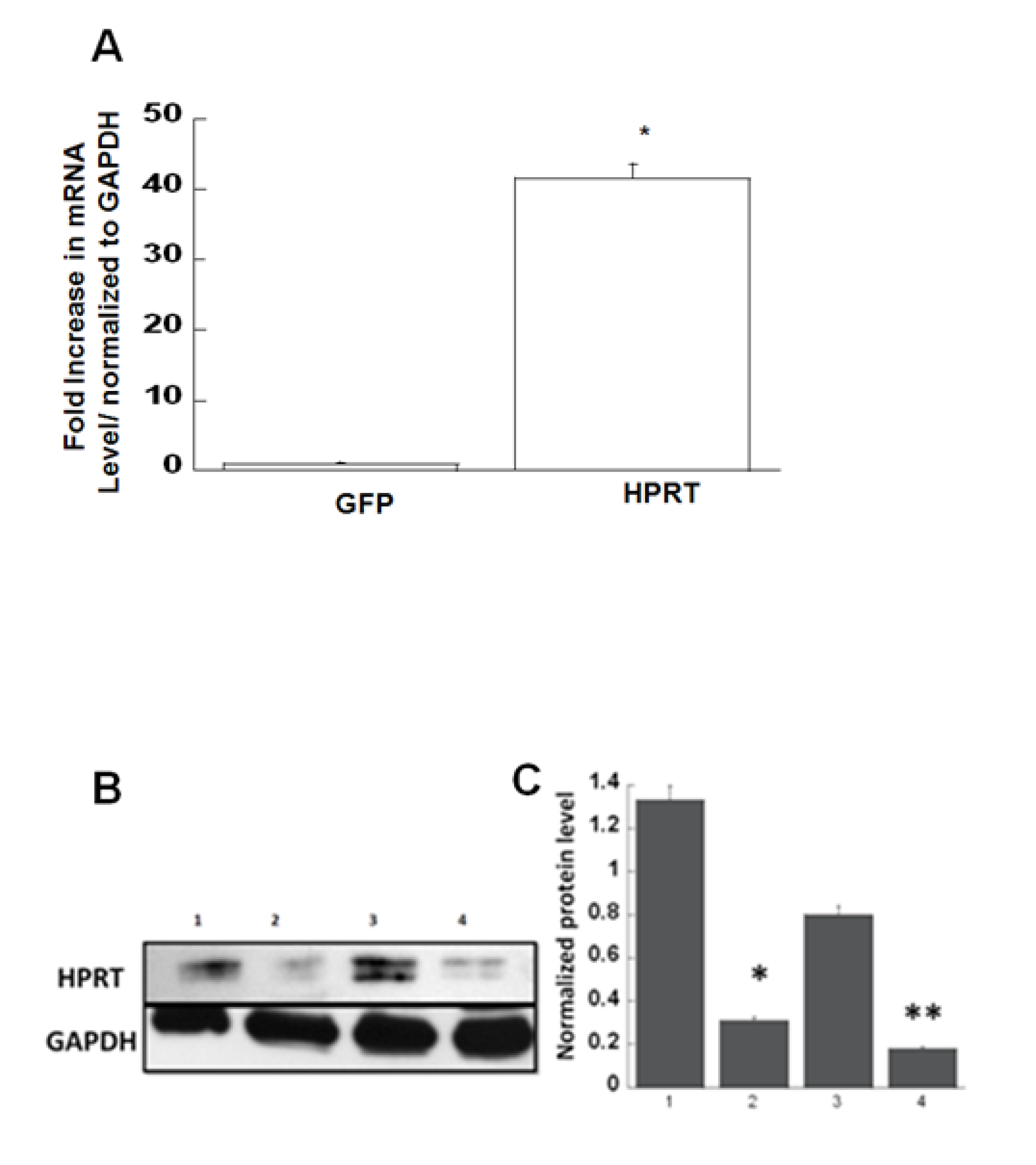

Supplement: Figure S7 — Gene expression profile of PDE10A in fibroblasts cells from normal (CTL) mildly (LNV) and severely (LND) affected HPRT-deficient patients. (A) ΔCT value of each category of patients, showing that LND subjects have significantly lower ΔCT than control (CTL); leading to significantly higher fold change in PDE10 mRNA level (B). (* p<0.05, ANOVA). (TIF) [file pone.0063333.s007.tif]

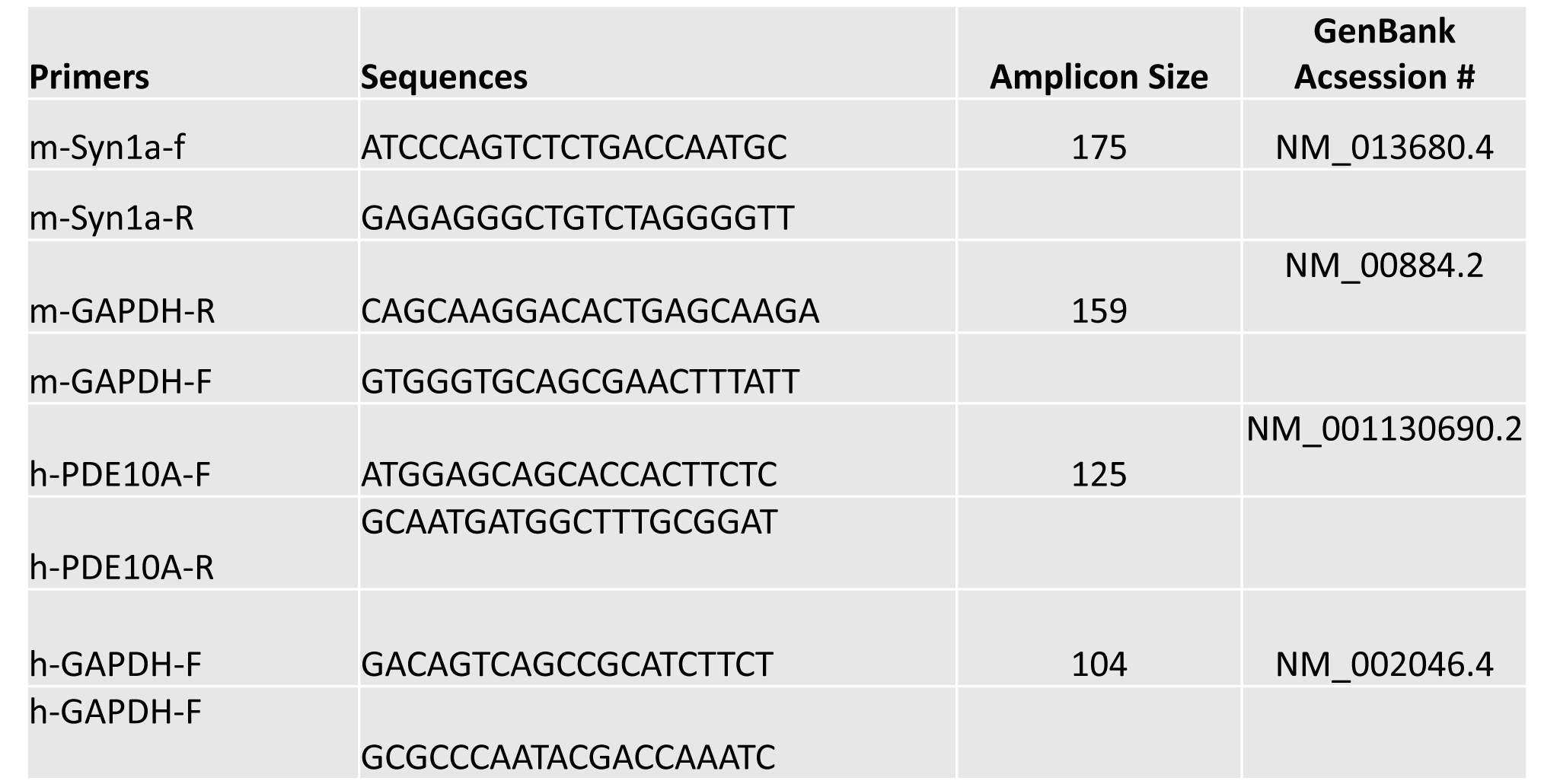

Supplement: Table S1 — List of primers. (TIF) [file pone.0063333.s008.tif]

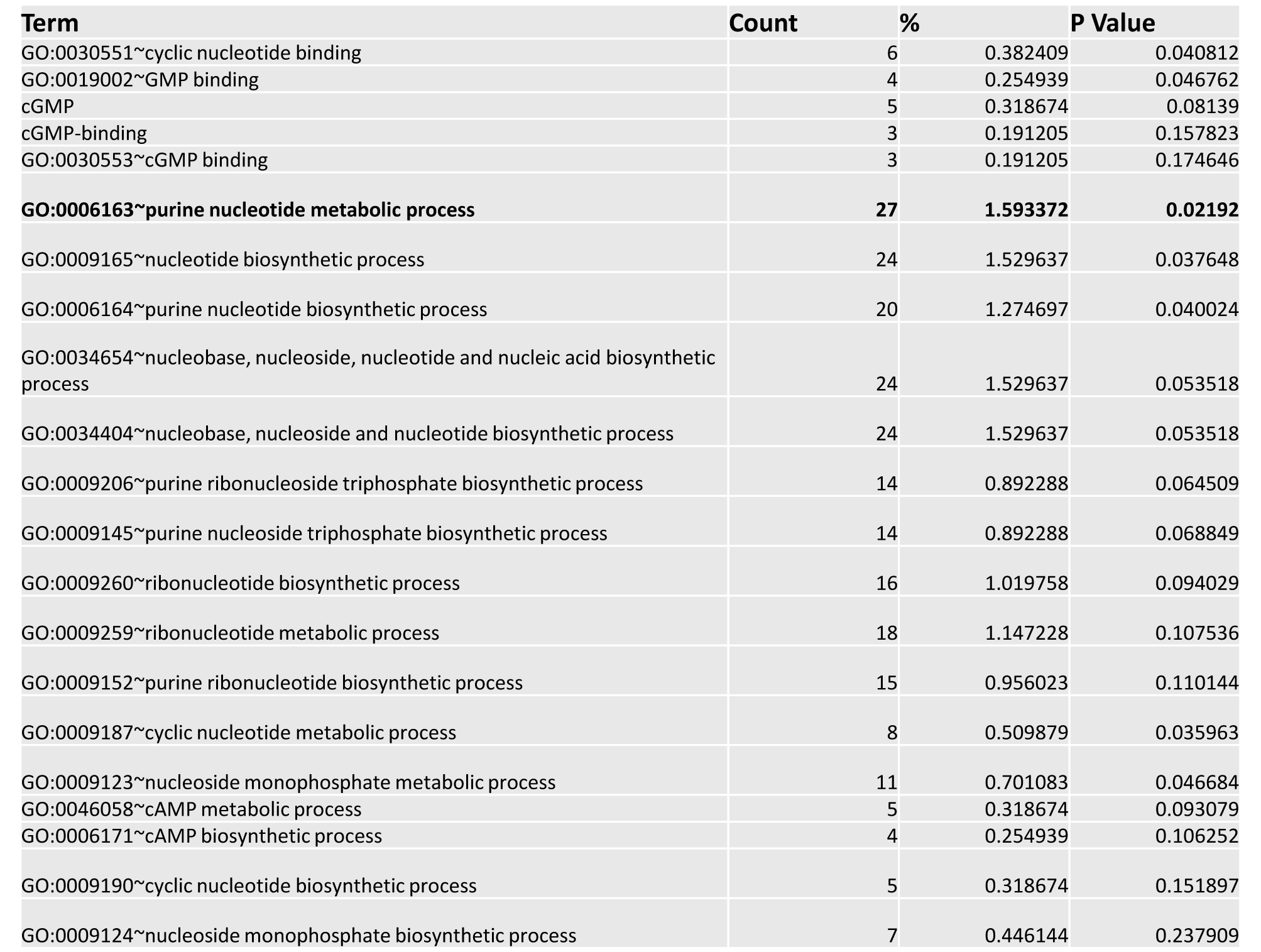

Supplement: Table S2 — Functional annotation clustering pertaining to purine pathways. The table includes GO terms related to “purine metabolism” derived from miR-181a potential target genes and selected from target-combo and targetScan database. GO terms were extracted using DAVID as previously described (Guibinga et al. 2012). Highlighted is the GO term “purine nucleotide metabolic process”. (TIF) [file pone.0063333.s009.tif]

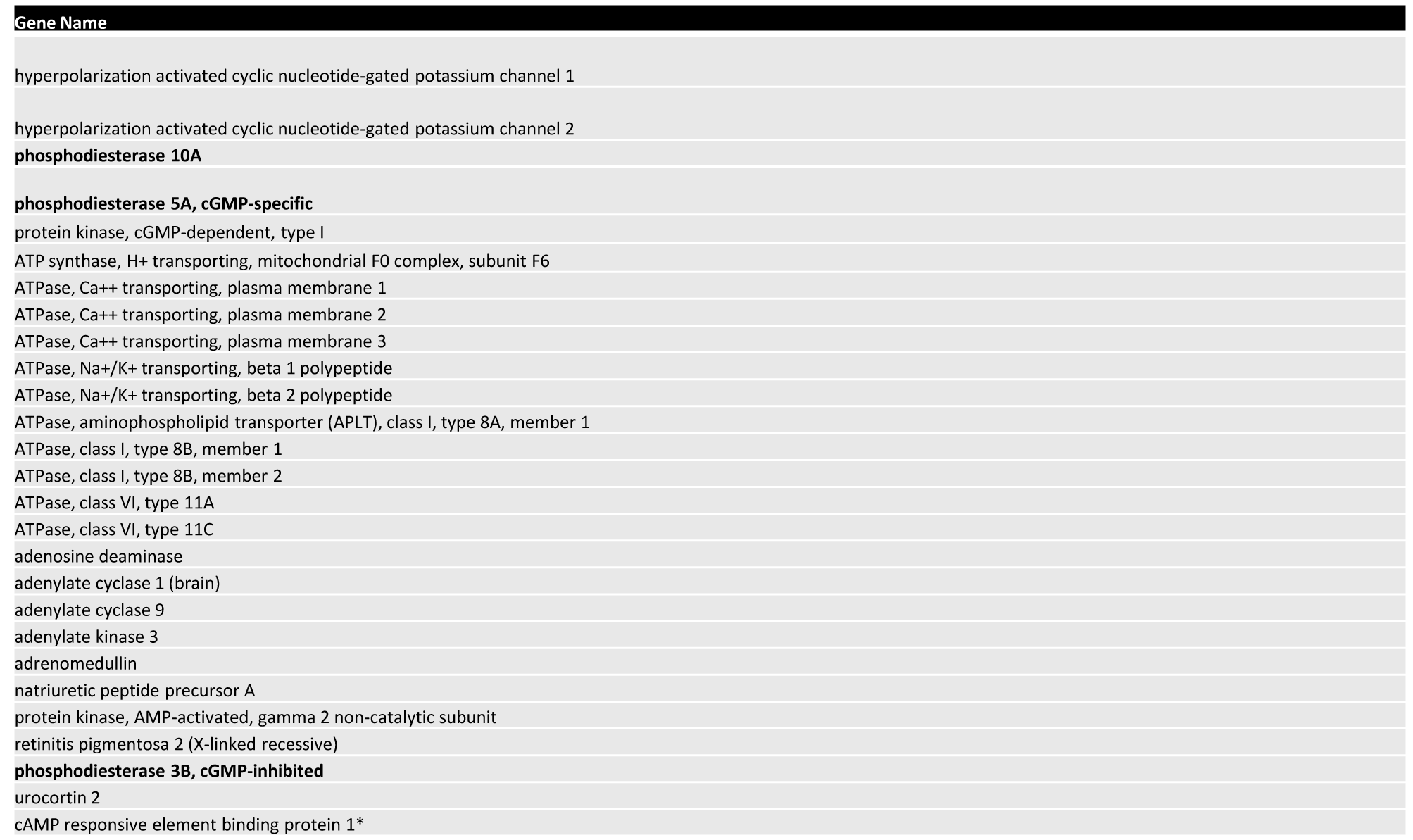

Supplement: Table S3 — List of potential miR-181a target genes derived from GO term related to “purine nucleotide metabolic process”. The table includes several genes known to regulate cAMP/PKA, such as PDE10. (TIF) [file pone.0063333.s010.tif]
